# Molecular mechanisms facilitating the initial kinetochore encounter with spindle microtubules

**DOI:** 10.1083/jcb.201608122

**Published:** 2017-06-05

**Authors:** Vanya Vasileva, Marek Gierlinski, Zuojun Yue, Nicola O’Reilly, Etsushi Kitamura, Tomoyuki U. Tanaka

**Affiliations:** 1 Centre for Gene Regulation and Expression, School of Life Sciences, University of Dundee, Dundee DD1 5EH, Scotland, UK; 2 Data Analysis Group, School of Life Sciences, University of Dundee, Dundee DD1 5EH, Scotland, UK; 3 Lincoln’s Inn Fields Laboratory, The Francis Crick Institute, London WC2A 3LY, England, UK

## Abstract

The initial kinetochore (KT) encounter with a spindle microtubule (MT; KT capture) is one of the rate-limiting steps in establishing proper KT–MT interaction during mitosis. KT capture is facilitated by multiple factors, such as MT extension in various directions, KT diffusion, and MT pivoting. In addition, KTs generate short MTs, which subsequently interact with a spindle MT. KT-derived MTs may facilitate KT capture, but their contribution is elusive. In this study, we find that Stu1 recruits Stu2 to budding yeast KTs, which promotes MT generation there. By removing Stu2 specifically from KTs, we show that KT-derived MTs shorten the half-life of noncaptured KTs from 48–49 s to 28–34 s. Using computational simulation, we found that multiple factors facilitate KT capture redundantly or synergistically. In particular, KT-derived MTs play important roles both by making a significant contribution on their own and by synergistically enhancing the effects of KT diffusion and MT pivoting. Our study reveals fundamental mechanisms facilitating the initial KT encounter with spindle MTs.

## Abstract (precis)

The initial kinetochore (KT) encounter with a spindle microtubule (MT) is one of the rate-limiting steps in establishing proper KT–MT interaction during mitosis. This study reveals how multiple factors cooperate to facilitate the KT encounter with a spindle MT. In particular, it highlights the important roles of KT-derived MTs in this process.

## Introduction

To maintain genetic integrity, eukaryotic cells must segregate their chromosomes to opposite spindle poles during anaphase before the cell division. Chromosome segregation is mainly driven by microtubules (MTs) that attach to kinetochores (KTs). The KT–MT interaction is established in a stepwise manner during the early stage of mitosis (prometaphase; Tanaka, 2010; Cheerambathur and Desai, 2014): KTs are initially loaded on the lateral side of an MT extending from a spindle pole (a spindle MT; Rieder and Alexander, 1990; Tanaka et al., 2005) and then tethered to the MT end (Maure et al., 2011; Shrestha and Draviam, 2013). Subsequently, sister KTs interact with MTs from opposite spindle poles (biorientation) after aberrant KT–MT interactions are removed (Tanaka et al., 2002; Kitamura et al., 2007). A delay in any of these steps could compromise the efficiency in establishing a proper KT–MT interaction. In particular, the initial KT–MT encounter often becomes a rate-limiting step in this process.

Several mechanisms are thought to facilitate the initial KT encounter with MTs extending from a spindle pole (Fig. S1 A): spindle MTs extend in various directions as if they are “searching” for KTs (search and capture; Kirschner and Mitchison, 1986). However, a random search-and-capture process does not completely explain the efficiency of the KT encounter with spindle MTs (Wollman et al., 2005). This is at least partly explained by the effect of the small GTPase Ran bound by GTP (Ran-GTP). Ran-GTP forms a concentration gradient around chromosomes, which “guides” the extension of a spindle MT toward a chromosome (Clarke and Zhang, 2008). These are thought to be long distance effects in the KT–MT encounter (Fig. S1 A). For example, a Ran-GTP gradient can only be formed over a long range (>5 µm) because of its rapid diffusion, and it cannot be formed in cells undergoing closed mitosis, e.g., budding yeast, whose nuclei contain a uniformly high Ran-GTP concentration (Carazo-Salas and Karsenti, 2003; Kaláb et al., 2006; Athale et al., 2008).

Over a short distance (up to 2–3 µm), KT encounter with spindle MTs is facilitated by KT diffusion as well as MT angular diffusion (pivoting) in which KTs and MTs move randomly and vigorously (Fig. S1 A; Wollman et al., 2005; Kitamura et al., 2007; Brun, 2011; Kalinina et al., 2013). The extent of KT and MT diffusion is subject to temperature and other aspects of the extracellular environment (Kalinina et al., 2013). Meanwhile, MTs can be generated not only at spindle poles but also at KTs (Khodjakov et al., 2003; Maiato et al., 2004; Kitamura et al., 2010). Such KT-derived MTs, which are short in both length and lifetime, grow and shrink in various directions. KT-derived MTs could subsequently interact with a spindle MT, and this event may facilitate KT encounter with a spindle MT. Indeed, the appearance of KT-derived MTs is correlated with a more efficient KT encounter with spindle MTs (KT capture; Kitamura et al., 2010). So far, however, there has been no evidence that KT-derived MTs promote KT capture because it has not been possible to specifically remove KT-derived MTs. For example, KT-derived MTs and rapid KT capture by spindle MTs may not be causatively related but may simply be facilitated by a common factor such as KT maturation. We revisit this issue by developing strategies for specifically removing KT-derived MTs, and we address the following questions: Do KT-derived MTs promote the KT capture? If so, what is the extent of this effect? Do KT-derived MTs, KT diffusion, and MT pivoting, involved in the short-range effect (Fig. S1 A), cooperate to facilitate KT encounter with spindle MTs? We address these questions using budding yeast as a model organism.

## Results

### Stu1 is required for Stu2 recruitment to KTs, which promotes MT generation at KTs

To address the role of KT-derived MTs in the initial KT encounter with spindle MTs, we aimed to abolish the generation of MTs by KTs without changing other KT functions or the dynamics of spindle MTs. We previously found that Stu2, the orthologue of vertebrate XMAP215/ch-TOG, localizes at KTs and plays a central role in generating MTs there before KT capture by spindle MTs (Kitamura et al., 2010). If we could remove Stu2 specifically at KTs, we should be able to specifically remove KT-derived MTs. However, Stu2 depletion itself is not suitable for this purpose because Stu2 is also required for MT generation at spindle poles in the nucleus (Tanaka et al., 2005).

**Figure 1.**
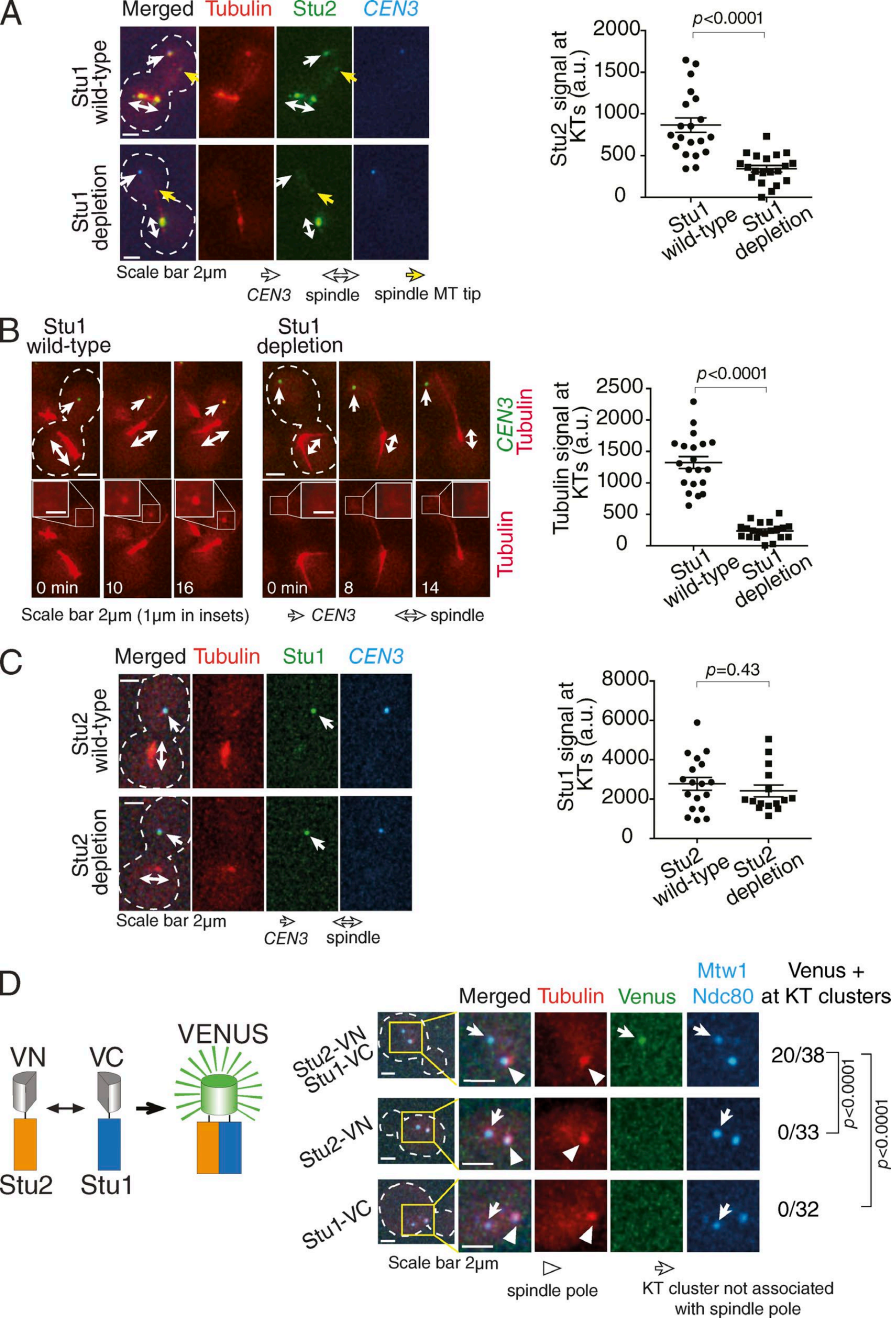
**Stu1 is required for Stu2 recruitment to KTs, which promotes MT generation at KTs.** (A) Stu1 depletion leads to a reduction of Stu2 signals at KTs. *STU1^+^* (T10699) and Stu1-aid (T10697) cells with *TIR1 P_GAL_-CEN3-tetOs TetR-3×CFP mCherry-TUB1 STU2-3×GFP P_MET3_-CDC20* were treated with mating pheromone for 2.5 h (to arrest in G1 phase) and released to fresh media with methionine (for Cdc20 depletion) in the presence of galactose (for *CEN3* inactivation). From 3 h after the release, auxin NAA was added to the media for 1 h to deplete Stu1-aid. Cells were then suspended in medium with methionine and with glucose (for *CEN3* reactivation) in the presence of NAA, and images were acquired every 2 min. Stu2 signals associated with *CEN3* were quantified at the time point before *CEN3* capture by a spindle MT (right; *n* = 20 in each condition), and representative images are shown (left). White dashed outlines show the shape of yeast cells. The p-value (two tailed) was obtained by an unpaired *t* test. Note that, after Stu1 depletion, Stu2 was significantly reduced at *CEN3* (white arrow) but not at the MT plus end (yellow arrow). (B) Stu1 depletion leads to the reduction of MT/tubulin signals at KTs. *STU1^+^* (T10594) and *stu1-aid* (T10501) cells with *TIR1 P_GAL_-CEN3-tetOs TetR-3×CFP GFP-TUB1 P_MET3_-CDC20* were treated, and their images were acquired as in A (left). Tubulin signals associated with *CEN3* were analyzed as in A (right; *n* = 20 in each condition). Insets show magnification of the area in which *CEN3* localizes. Glucose was added at time 0. (C) Stu2 depletion leads to a loss of MT/tubulin signal at KTs but does not change the amount of Stu1 there. *STU2^+^* (T10833) and *stu2-aid* (T10834) cells with *TIR1 P_GAL_-CEN3-tetOs TetR-3×CFP mCherry-TUB1 STU1-2×GFP P_MET3_-CDC20* were treated, and their images were acquired as in A. Stu1 signals associated with *CEN3* were analyzed as in A (*n* = 18 and 15 for Stu2 wild-type and Stu2 depletion, respectively). Note that after depletion of Stu2, KT-derived MTs were abolished and MT signals were reduced on the spindle, as shown in this example of Stu2 depletion and previously (Kitamura et al., 2010). a.u., arbitrary unit. (D) Stu1 and Stu2 are closely associated at noncaptured KTs. The diagram illustrates that a close association between Stu1-VC and Stu2-VN leads to the generation of Venus signals. *STU2-VN STU1-VC* (T12563), *STU2-VN* (T12633), and *STU1-VC* (T12731) cells with *mCherry-TUB1 MTW1-3×CFP NDC80-3×CFP* (where *VN* and *VC* are N- and C-terminal fragments of Venus fluorescent protein) were treated with mating pheromone for 3 h and then released into fresh media supplemented with nocodazole. 60 min after the release, images were acquired. Numbers of bright KT clusters (not associated with a spindle pole; denominators) and those with Venus signals (numerators) are shown on the right. P-values (two tailed) were obtained by Fisher’s exact test.

To identify the factors required for Stu2 recruitment to KTs, we used an engineered assay system in which KT assembly was delayed on a chosen centromere by transcription from an adjacently inserted promoter (Tanaka et al., 2005). This increased the distance between the centromere and the mitotic spindle. We then shut off the adjacent promoter, allowed KT assembly, and observed KTs before their capture by spindle MTs (centromere reactivation assay). We often detected MT/tubulin signals at KTs; the signal was often punctate, containing multiple short MTs and occasionally one or two longer MTs (Kitamura et al., 2010). Stu1, the orthologue of vertebrate CLASP, is an MT-associated protein that also localizes at KTs (Fig. S1 B; Pasqualone and Huffaker, 1994; Ortiz et al., 2009). To address any possible relationship between Stu1 and Stu2 localization at KTs, we tagged Stu1 with an auxin-induced degron (*aid*) and depleted it by adding auxin (Fig. S1 C, top; Nishimura et al., 2009). Intriguingly, Stu1 depletion led to considerable reduction of Stu2 and MT/tubulin signals at KTs that were not yet captured by spindle MTs (noncaptured KTs; Fig. 1, A and B; and Fig. S1 D). The inverse was not the case, however; i.e., Stu2 depletion abolished MT signals but did not change Stu1 localization at KTs (Figs. 1 C and S1 C, bottom). These data suggest that Stu1 is recruited to noncaptured KTs and in turn recruits Stu2 there.

Is Stu1 directly involved in Stu2 recruitment to KTs? To address this, we tested whether Stu1 and Stu2 associate closely with each other. We used a bimolecular fluorescence complementation assay (Kerppola, 2008) in which Stu2 and Stu1 were fused with the N and C terminus halves, respectively, of the Venus fluorescent protein (Stu2-VN and Stu1-VC). If Stu1 and Stu2 associated closely (<10 nm), a complete Venus protein was generated to give a fluorescence signal (Fig. 1 D, left). We observed clusters of noncaptured KTs, which were generated by treating cells with the MT polymerization inhibitor nocodazole (Richmond et al., 2013). The KT clusters gave a higher chance of detecting potential Venus signals at KTs than would an isolated KT. Note that, in these conditions, Stu1 and Stu2 localized on clustered noncaptured KTs, and Stu2 localization there was dependent on Stu1 (Fig. S1, E and F) as in Fig. 1 A. In cells with Stu1-VC and Stu2-VN, we detected Venus signals at clustered noncaptured KTs (Fig. 1 D, right; and Fig. S1 G), indicative of close association between Stu1 and Stu2 at KTs. Collectively, our results suggest that Stu1 associates closely with Stu2 to promote Stu2 recruitment to KTs, which then facilitates MT generation at KTs.

### Once Stu2 accumulates at the KT, it no longer requires Stu1 for MT generation

**Figure 2.**
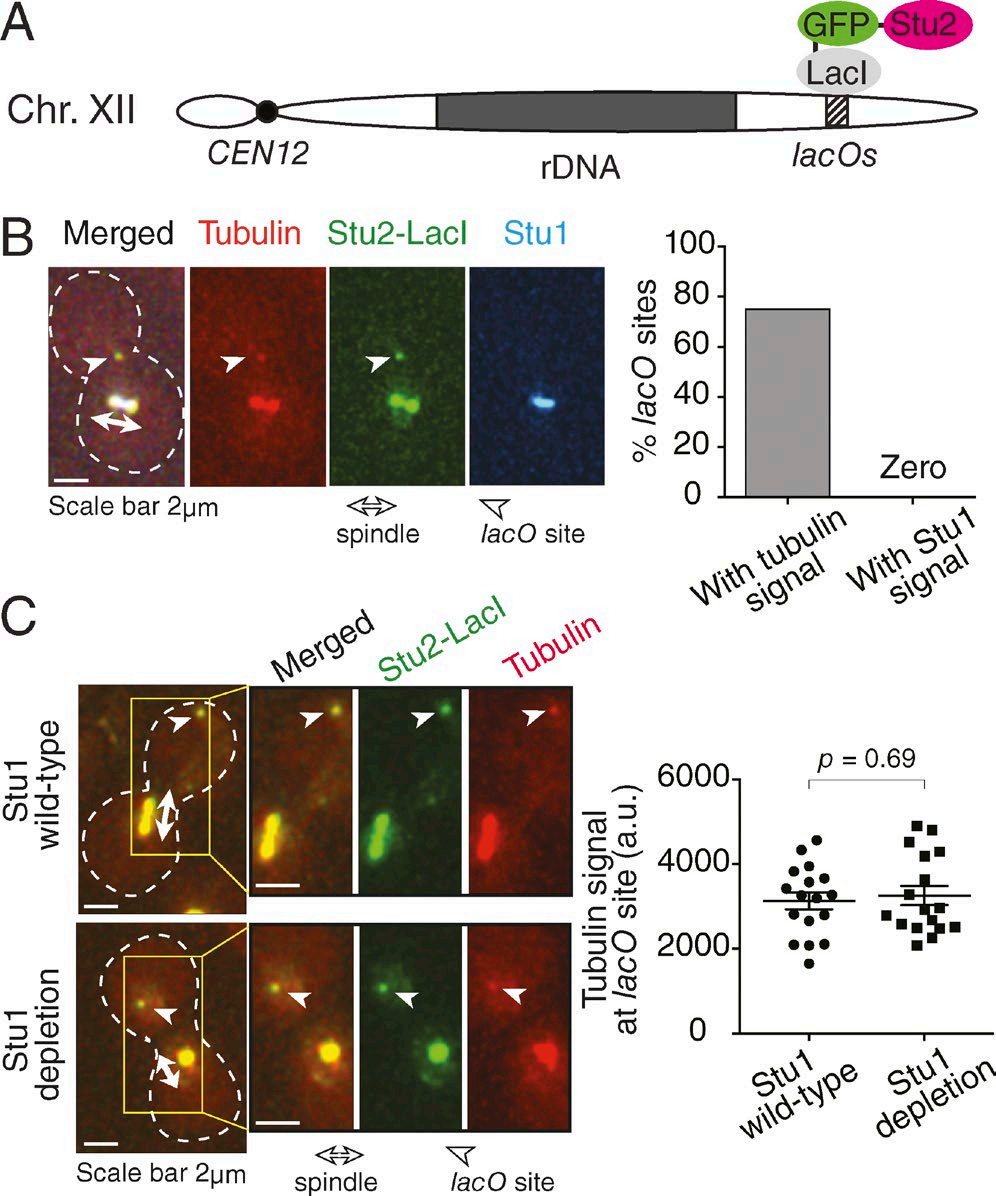
**Stu2 accumulation is sufficient for MT generation, and Stu1 is not required for it.** (A) Diagram showing that Stu2-GFP-LacI is tethered at *lacO*s at a chromosome (Chr) arm locus. rDNA, ribosomal DNA. (B) The Stu2-GFP-LacI–tethered site is associated with MT/tubulin signals but not with Stu1 signals. *P_GALS_-STU2-GFP-LacI REC102:lacO*s *STU1-4×mCherry CFP-TUB1 P_MET3_-CDC20* (T11037) cells were treated with mating pheromone for 2.5 h and then released into fresh media supplemented with raffinose and methionine. From 3 h after the release, galactose was added to the media for 1 h to express Stu2-GFP-LacI, and images were acquired. White dashed outline shows the shape of a yeast cell. Graph shows the percentages of *lacO*s sites with tubulin and Stu1 signals. *n* = 28. Note that in spite of the absence of Stu1 at the Stu2-GFP-LacI–tethered site, Stu1 still showed normal localization on the spindle. (C) MT/tubulin signals appear at the Stu2-GFP-LacI–tethered site in both the presence and absence of Stu1. *STU1^+^* (T11722) and *stu1-aid* (T11686) cells with *TIR1 P_GALS_-STU2-GFP-LacI REC102:lacO*s *CFP-TUB1 P_MET3_-CDC20* cells were treated as in B, except galactose was also added to the media 2 h after the release. From 3 h after the release, auxin NAA was added to the media for 1 h (to deplete Stu1-aid), and images were acquired. Tubulin signals associated with the *lacO*s site were quantified (graph; *n* = 17 in each condition). The p-value (two tailed) was obtained by an unpaired *t* test. Note that the collapsed bipolar spindle (left, bottom) suggests that Stu1 was indeed depleted in this condition (Yin et al., 2002). a.u., arbitrary unit.

Stu1 possesses TOG domains that can potentially bind tubulins (Al-Bassam and Chang, 2011), and thus it may help Stu2 to generate MTs at KTs. Alternatively, Stu1 may not be involved in MT generation at KTs after recruiting Stu2 there. To test these possibilities, Stu2 was fused with GFP and a *lac* repressor, LacI (Stu2-GFP-LacI) and was tethered to an array of *lac* operators (*lacO*s) integrated on a chromosome arm locus (Fig. 2 A). We previously showed that such an ectopic Stu2 accumulation (with a similar level of accumulation to that at the KT) leads to MT generation there (Kitamura et al., 2010). When we visualized Stu1 in an equivalent experiment, we could not detect Stu1 at the Stu2-tethered site (Fig. 2 B). We also repeated the Stu2-tethering experiments after Stu1 depletion. MTs were still generated at the Stu2-tethered site after Stu1 depletion (Fig. 2 C), and this was the case even when the Stu2 level at the tethering site was similar to that at the KT (Fig. S2 A). Therefore, Stu2 accumulation is sufficient for MT generation and does not require Stu1 function. Based on this, we reason that, once Stu1 recruits Stu2 to KTs, Stu1 is no longer required for MT generation at KTs. Our previous result also suggested that γ-tubulin is not required for MT generation at KTs (Kitamura et al., 2010). Stu2 orthologues show MT nucleation activity in vitro (Wieczorek et al., 2015), and therefore, Stu2 may not require any other factors for MT generation.

We next studied the outcome of Stu1 accumulation at a chromosome arm locus by tethering Stu1-GFP-LacI, instead of Stu2-GFP-LacI, to the *lacO*s (Fig. S2 B). We could not detect MT/tubulin signals at the Stu1-tethered site in contrast to the Stu2-tethering experiment (Fig. S2 C). In a control experiment, MT/tubulin signals were detected at KTs in cells expressing Stu1-GFP-LacI as a sole Stu1 protein (Fig. S2 D), suggesting that Stu1-GFP-LacI is functional and able to generate MTs at KTs. A corollary is that Stu1 is insufficient to generate MTs at KTs. Stu1 is presumably insufficient to recruit Stu2 to KTs and, in addition to Stu1, other KT components are required for the Stu2 recruitment. Consistent with this, it was reported that mutation of Ndc80 complex components abolishes KT localization of Stu2 but not of Stu1 (Ma et al., 2007; Kitamura et al., 2010; Miller et al., 2016).

### Stu1 depletion leads to both a lack of KT-derived MTs and a delay in KT capture by spindle MTs

**Figure 3.**
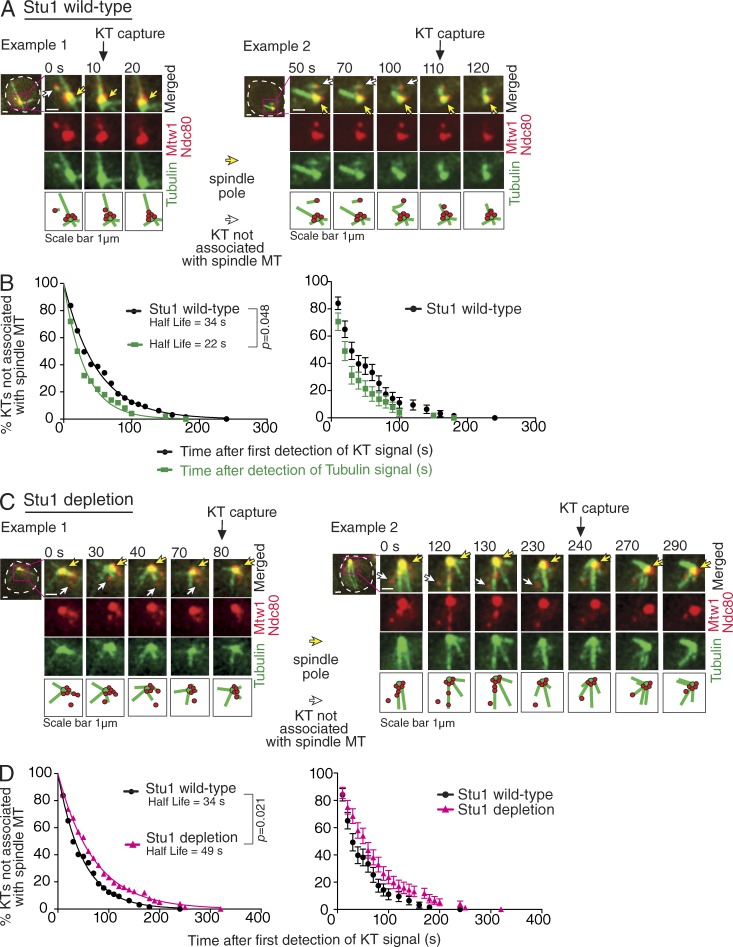
**After Stu1 depletion, KT-derived MTs are abolished, and KT capture by spindle MTs is delayed in physiological conditions.** (A) Two examples of KT capture by spindle MTs in *STU1* wild-type cells. *STU1^+^ MTW1-4×mCherry NDC80-4×mCherry YFP-TUB1* cells (T11242) were treated with a mating pheromone for 3 h and released to fresh media. 30 min before the release, rapamycin was added to the media (it was present after the release as well). From 20 min after the release, images were acquired every 10 s. The first time point when a noncaptured KT was detected is denoted as time 0. On the left, a KT-associated tubulin signal was visible at 0 s, and the KT was captured by a spindle MT at 10 s. On the right, an MT extended from the KT at 50–100 s, and the KT was captured by a spindle MT at 110 s. White dashed outlines show the shape of yeast cells. KT capture was discerned when an overlap of a KT signal with spindle MTs was followed by KT motion toward a spindle pole. (B) The percentage of noncaptured KTs (100% at time 0; time 0 is defined as in A) was plotted and fitted to a simple exponential decay curve in wild-type cells (left, black dots and line) based on the result in Fig. S3 A, Stu1 wild-type (*n* = 60). A KT-associated MT/tubulin signal appeared at a single or consecutive time points, and the first time point of each appearance was defined as the new time 0 for replotting the percentage of noncaptured KTs (left, green squares and line). Half-lives were calculated based on fitted exponential decay curves. (C) Two examples of the KT capture by spindle MTs with Stu1 depletion. *stu1–anchor-away MTW1-4×mCherry NDC80-4×mCherry YFP-TUB1* cells (T11230) were treated, and images were acquired as in A. Time 0 is defined as in A. A KT was observed first at time 0 and captured by a spindle MT at 80 s (left) and 240 s (right), and no tubulin signal was associated with noncaptured KTs. (D) The percentage of noncaptured KTs in Stu1-depleted cells (100% at time 0; time 0 is defined as in A) was plotted and fitted to a simple exponential decay curve (left, magenta triangles and line) based on the result in Fig. S3 A, Stu1 depletion (*n* = 74). The result for Stu1 wild-type cells in A is shown for comparison (black dots and line). Error bars represent a standard error of proportion for the data points (right). P-values (two tailed) were obtained by log-rank tests for survival curves.

So far, we used the centromere reactivation assay, nocodazole treatment, and protein tethering to *lacO*s in our study. Next, we investigated initial KT capture by spindle MTs in more physiological conditions. In physiological conditions, KTs are attached to spindle MTs during most of the cell cycle in budding yeast (Winey and O’Toole, 2001). Upon centromere DNA replication, KTs are at least partially disassembled, leading to detachment of centromeres from MTs. KTs are reassembled within 1–2 min (Kitamura et al., 2007) and interact with MTs again (Fig. S3 A, Stu1 wild-type). MT/tubulin signals are often found at noncaptured KTs. When an MT extends from a KT, such a KT-derived MT often interacts with a spindle MT, as in the centromere reactivation assay (Fig. 3 A; Kitamura et al., 2010). As we previously reported (Kitamura et al., 2010), the percentage of noncaptured KTs declines with the time elapsed from appearance of KT signals (defined as time 0), and this decline was a good fit to a simple exponential decay curve (half-life of 34 s; Fig. 3 B, black line). As expected from the nature of a simple exponential decay curve, very similar curves were obtained when we replotted the decay curve using randomly selected points as new time 0 (Fig. S3 B, dashed lines). To address whether KT-derived MTs affect the timing of KT capture, we redefined the first appearance of MT/tubulin signals at KTs as a new time 0 and replotted the decline of noncaptured KTs. Their number declined more rapidly (Fig. 3 B, green squares and line) than the original decay curve (black dots and line). This suggests that appearance of KT-derived MTs is correlated with more rapid KT capture, consistent with our previous finding (Kitamura et al., 2010).

However, this correlation may not necessarily reflect a direct causative relationship between KT-derived MTs and KT capture. For example, a common factor (e.g., KT maturation) may promote both KT-derived MTs and efficient KT capture. Such a causative relationship could be tested by the depletion of Stu1, which reduces MT signals at KTs (Fig. 1) and may make KT capture less efficient. We depleted Stu1 in the nucleus by anchoring Stu1 to ribosomes (Fig. S3 C; Haruki et al., 2008) and observed KT appearance and capture by spindle MTs (Figs. 3 C and S3 A, Stu1 depletion). After Stu1 depletion in the nucleus, only a few MT/tubulin signals were found at noncaptured KTs, as expected (green in Fig. S3 A, Stu1 depletion). Then, we plotted the percentage of noncaptured KTs with time (Fig. 3 D, magenta triangles and line); i.e., time elapsed from appearance of KT signals (time 0). The decline of noncaptured KTs was compared in Stu1-depleted (Fig. 3 D, magenta) and *STU1* wild-type control cells (Fig. 3 D, black). These results suggest that Stu1 depletion leads to both a lack of KT-derived MTs and a delay in KT capture by spindle MTs (half-life, 49 s). This result is consistent with a previous finding that Stu1 facilitates KT capture by spindle MTs after nocodazole treatment and washout (Ortiz et al., 2009). Furthermore, we repeated the experiments in Fig. 3; i.e., we acquired an independent dataset, analyzed it in the same way as Fig. 3, and reached the aforementioned conclusion (Fig. S3 D).

### Evidence that KT-derived MTs could facilitate KT capture by spindle MTs

**Figure 4.**
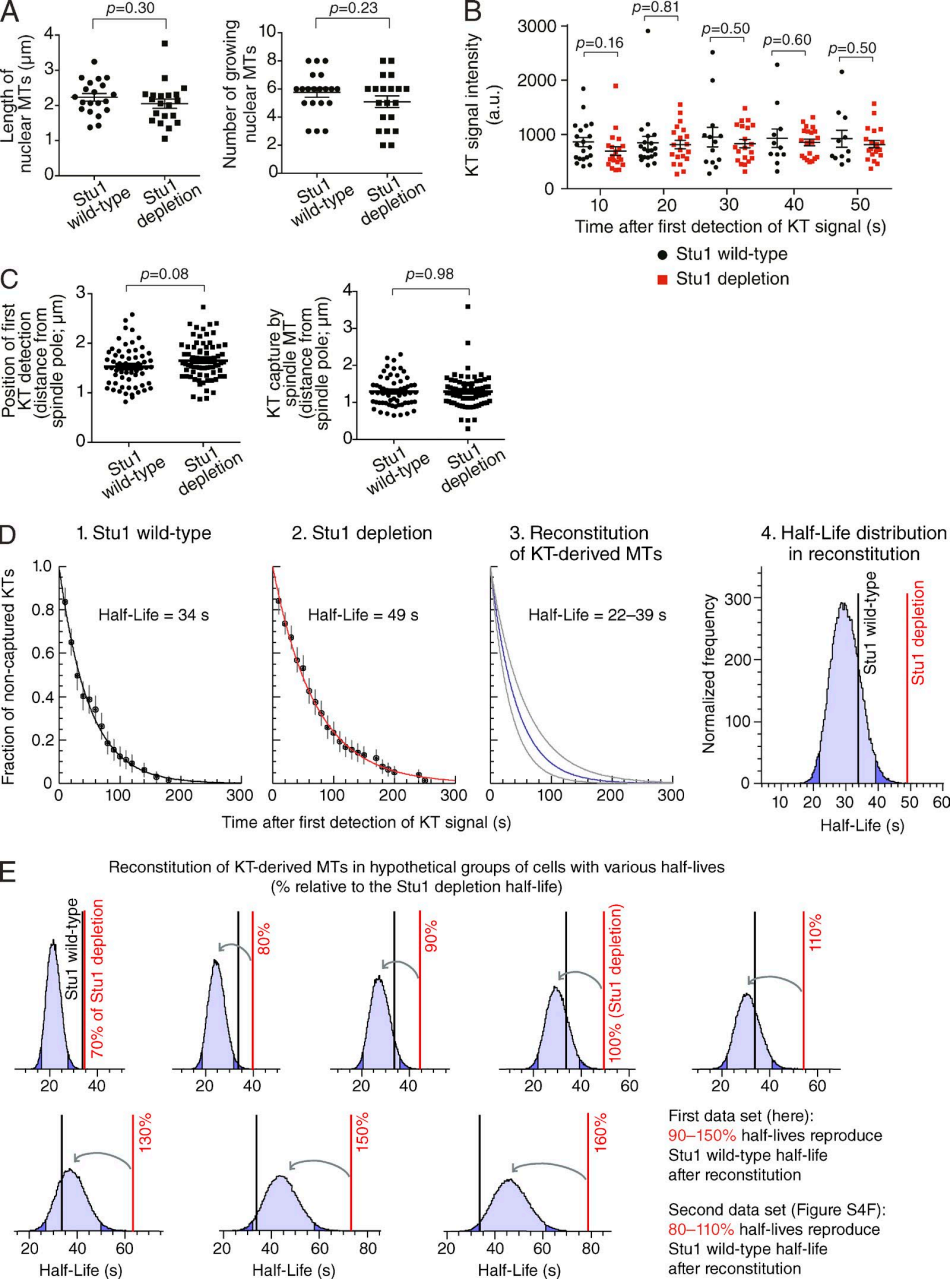
**Implication that KT-derived MTs facilitate KT capture by spindle MTs.** (A) There is no significant difference in the length or number of nuclear MTs in Stu1 wild-type and Stu1-depleted cells. *STU1^+^* (T11802) and *stu1–anchor-away* (T11766) cells with *NIC96-4×mCherry GFP-TUB1* cells were treated, and images were acquired as in Fig. 3 A. Nic96 signals were used to identify the periphery of nucleus. The p-value (two tailed) was obtained by an unpaired *t* test. The maximum length (left) and the maximum number (right) of nuclear MTs, which were observed during 5 min, are plotted in individual cells (*n* = 20 in each condition). (B) There is no significant difference in KT signal intensity between Stu1 wild-type and Stu1-depleted cells. *STU1^+^* (T11242) and *stu1–anchor-away* (T11230) cells with *MTW1-4×mCherry NDC80-4×mCherry YFP-TUB1* cells were treated and analyzed as in A. The intensity of the KT signal is plotted at each time point (time after the first appearance of the KT signal) in individual cells. *n* = 20 at each time point and in each condition, except for 30 s (*n* = 13), 40 s (*n* = 11), and 50 s (*n* = 11) in Stu1 wild-type. a.u., arbitrary units. (C) There is no significant difference in the position of the KT appearance or capture by spindle MTs between Stu1 wild-type and Stu1-depleted cells. T11242 and T11230 cells were analyzed as in B. The distances from a spindle pole of first KT appearance (left) and KT capture by spindle MTs (right) are plotted for individual events (*n* = 60 and 74 for Stu1 wild-type and Stu1 depletion, respectively). The mean ± standard error is shown in each group of cells. (D) Computational reconstitution of KT-derived MTs in Stu1-depleted cells generated decline curves of noncaptured KTs, which are consistent with Stu1 wild-type cells. Graphs 1 and 2 show the decline curve of noncaptured KTs observed in vivo in Stu1 wild-type and Stu1-depleted cells, respectively (based on Fig. S3 A). Lines show best-fitting simple exponential decay curves, and error bars represent standard errors of proportion. Graph 3 shows the range of decline curves of noncaptured KTs based on results from 100,000 runs of computational reconstitution of KT-derived MTs. In each run, KT-derived MTs were added to a group of Stu1-depleted cells (see Fig. S4 D). The gray curves represent the central 95% of all simulation runs. The corresponding range of half-lives is also shown. Graph 4 shows the distribution of half-lives of noncaptured KTs after the computational reconstitution of KT-derived MTs. Light blue areas represent the range within the 95% CI, and dark blue areas show the range outside of it. Black and red vertical lines show the half-lives of Stu1 wild-type and Stu1-depleted cells, respectively. See Fig. S4 E for analyses of the second dataset. (E) Stu1-depleted cells show a delay in KT capture whose extent falls within a relatively narrow range expected from a lack of KT-derived MTs. KT-derived MTs were computationally reconstituted (100,000 runs in each condition) in hypothetical groups of cells where half-lives of noncaptured KTs (red vertical lines) are 70–160% of the half-life (49 s) in Stu1-depleted cells observed in vivo. Light blue areas, dark blue areas, and black vertical lines are as in D. The y axis of each graph shows normalized frequency. See Fig. S4 F for analyses of the second dataset.

As shown in the previous section, Stu1 depletion led to both a lack of KT-derived MTs and a delay in KT capture. A lack of KT-derived MTs may be a reason for the delay in KT capture in Stu1-depleted cells. Alternatively, Stu1 may not only promote KT-derived MTs but may also promote KT assembly or change the dynamics of spindle MTs to facilitate efficient KT capture. To test this alternative possibility, we compared the length of spindle MTs, the number of growing spindle MTs, and the intensity of tubulins on the whole spindle in Stu1-depleted and *STU1* wild-type control cells (Figs. 4 A and S4 A). We also compared the intensity of KTs between these two conditions (Figs. 4 B and S4, B and C). Furthermore, we investigated the position (distance from a spindle pole) of KT appearance and KT capture by spindle MTs (Fig. 4 C). There was no significant difference in any of these parameters between Stu1-depleted and *STU1* wild-type cells, suggesting that Stu1 is not involved in KT assembly or MT dynamics in this context. Note that Stu1 does not localize at the MT plus end in budding yeast (Fig. S1 B; Pasqualone and Huffaker, 1994), consistent with there being no involvement of Stu1 in the growth of spindle MTs. Stu1 is required for the formation of a bipolar spindle (Yin et al., 2002), but this was not relevant to the present study because the bipolar spindle is formed only after all KTs are captured and collected to the vicinity of a spindle pole (Lim et al., 1996; Kitamura et al., 2007). Thus, as far as we investigated, there was no evidence that Stu1 depletion changes KT assembly or dynamics of spindle MTs.

Then, is a lack of KT-derived MTs actually a major reason for the delay in KT capture by spindle MTs in Stu1-depleted cells? To address this, we attempted computational reconstitution of KT-derived MTs in Stu1-depleted cells by taking the following steps (Fig. S4 D): (a) we investigated the properties of KT-derived MTs in *STU1* wild-type cells; (b) based on these properties, we then stochastically generated KT-derived MTs in Stu1-depleted cells by computation; and (c) from this dataset, we obtained a new decline curve of noncaptured KTs and compared it with the decline curve in *STU1* wild-type cells (Fig. 4 D, graph 1). For step a, we quantified the frequency of appearance of KT-derived MTs, their duration (lifetime), and their success rate of assisting KT capture in *STU1* wild-type cells (Fig. S4 D). If KT capture immediately followed the presence of KT-derived MTs, we interpreted it as a successful assistance of KT capture by KT-derived MTs. In step b, if a successful KT-derived MT with a certain lifetime was added to an event in a Stu1-depleted cell, we advanced the time point of KT capture accordingly (Fig. S4 D, inset 2). In this way, we produced a new decline curve of noncaptured KTs in Stu1-depleted cells with computer-generated KT-derived MTs and repeated this procedure to draw decline curves many times (Fig. 4 D, graph 3). We found that the half-life observed in *STU1* wild-type cells in vivo was within the 95% confidence interval (CI) of half-lives of the reconstituted decline curves (Fig. 4 D, graph 4). We repeated the same analysis using the second independent dataset acquired for Fig. S3 D and obtained a similar result (Fig. S4 E). Thus, the computational reconstitution of KT-derived MTs in Stu1-depleted cells can reproduce the efficiency of KT capture by spindle MTs observed in *STU1* wild-type cells.

To analyze these results further, we next reconstituted KT-derived MTs in the aforementioned way, but in hypothetical groups of cells where KT capture was more rapid or slower than in Stu1-depleted cells (Fig. 4 E). When hypothetical groups had 90–150% half-lives of the Stu1 depletion half-life (but not when they had shorter or longer half-lives than this range), they could reproduce a *STU1* wild-type half-life (i.e., the *STU1* wild-type half-life was within the 95% CI of the half-lives obtained) after reconstitution of KT-derived MTs. Meanwhile, in the second dataset acquired for Fig. S3 D, when they had 80–110% half-lives, they could reproduce the *STU1* wild-type half-life (Fig. S4 F). These results suggest that only a relatively narrow range of half-lives, including the Stu1 depletion half-life, can reproduce the *STU1* wild-type half-life after computational addition of KT-derived MTs. In other words, Stu1-depleted cells showed a delay in KT capture, whose extent falls within a relatively narrow range expected from a lack of KT-derived MTs. Collectively, these results are consistent with a lack of KT-derived MTs being a major reason for a delay in KT capture by spindle MTs in Stu1-depleted cells. If this notion is correct, KT-derived MTs facilitate KT capture by spindle MTs.

KT-derived MTs are relatively short in length (Figs. 1 B and 3 A; Kitamura et al., 2010). So, if KT-derived MTs indeed facilitate KT capture by spindle MTs, this would happen only in close proximity to the spindle MTs. Therefore, immediately after KTs come close to spindle MTs, the rate of KT capture by spindle MTs might be higher in the presence of KT-derived MTs than in their absence. This was indeed the case (Fig. S4 G), and this result is also consistent with KT-derived MTs facilitating KT capture by spindle MTs when KTs closely approach spindle MTs.

### How multiple factors facilitate the initial KT encounter with spindle MTs: redundancy and synergy of their contributions

**Figure 5.**
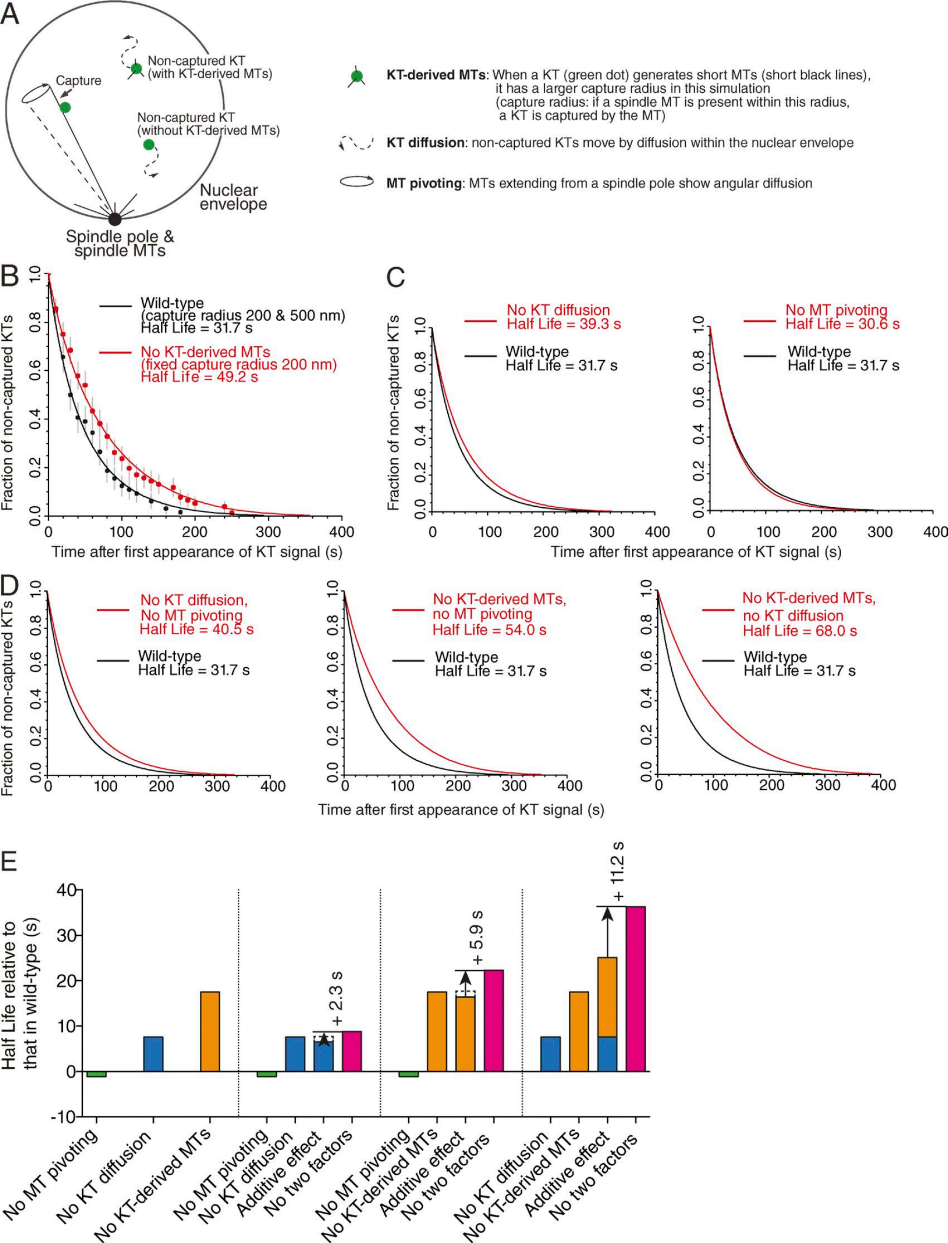
**Computational simulation of the initial KT encounter with spindle MTs reveals redundancy and synergy between multiple facilitating factors.** (A) Diagram outlines computer simulation that recapitulates the initial KT–MT interaction in budding yeast (Gandhi et al., 2011). Upon *CEN* replication, KTs disassemble and *CEN*s move away from a pole (Kitamura et al., 2007). KTs are then reassembled on *CEN*s (green dots; noncaptured KTs). They are subsequently captured by MTs that extend from a spindle pole. We simulate how efficiently KTs are captured by MTs, i.e., how rapidly the fraction of noncaptured KTs decreases. The effects of the following three factors are analyzed (right): (1) KT-derived MTs, (2) KT diffusion, and (3) MT pivoting. (B) Computer simulation recapitulates kinetics of KT capture by spindle MTs. Black and red dots show the observed fraction of noncaptured KTs along the time course in Stu1 wild-type and Stu1-depleted cells, respectively (see Figs. 3 and S3 A; time 0 is as in Fig. 3, A and C; error bars are as in Fig. 4 D). Black and red lines show the results of spatiotemporal simulations (100,000×) in wild-type cells (a larger and smaller KT capture radius interchange) and in the absence of KT-derived MTs (always a smaller capture radius). A standard error of the fraction of noncaptured KTs is <0.001 (see the Spatiotemporal simulation of KT–MT interaction section of Materials and methods). A half-life obtained from each simulation is shown in each condition (a standard error is <0.3 s). (C) Kinetics of KT capture by spindle MTs in the absence of KT diffusion or MT pivoting. The decline of noncaptured KTs is plotted in each condition (red line) based on the spatiotemporal simulations. The decline in wild type is also shown as a control (black line). Time 0 is as in B. Errors in fractions and half-lives are as in B. (D) Kinetics of KT capture by spindle MTs in the absence of two factors. The decline of noncaptured KTs is plotted when two factors are missing (red line) based on the spatiotemporal simulations. The decline in wild type is also shown as a control (black line). Time 0 is as in B. Errors in fractions and half-lives are as in B. (E) Redundancy and synergy in facilitating KT capture by spindle MTs between KT-derived MTs, KT diffusion, and MT pivoting. Graph shows the half-life of noncaptured KTs (see B, C, and D) in the absence of each factor (bars in green, blue, and orange) or two factors (bars in magenta) relative to the half-life in the wild-type condition. An “additive effect” is estimated by adding a half-life relative to that in wild-type in the absence of each factor; for example, the half-life with no KT diffusion (blue bar) and with no KT-derived MT (orange bar) is 7.6 s and 17.5 s, respectively, relative to that in wild type; thus, the additive effect is 7.6 + 17.5 = 25.1 s. An arrow indicates the difference between an additive effect and an observed half-life without two factors; for example, when KT diffusion and KT-derived MTs are missing, the observed half-life is 36.3 s (relative to that in wild type), which is 11.2 s (36.3 – 25.1 = 11.2) more than the corresponding additive effect. When this difference shows a positive value, it is defined as a synergistic effect. Errors in half-lives are as in B.

As discussed in the Introduction, it is thought that multiple factors facilitate KT capture by spindle MTs; KT diffusion, MT pivoting, and KT-derived MTs could facilitate this process over short distances (Figs. 5 A, inset; and Fig. S1 A). What is the relative contribution of each factor in shortening KT capture time? Do these factors cooperate to facilitate KT capture, and if so, how? To address these questions, we chose to use spatiotemporal computer simulation of the KT–MT interaction (Gandhi et al., 2011) because it is difficult to specifically switch off KT diffusion or MT pivoting in live-cell imaging. We computationally simulated KT assembly and subsequent capture by an MT extending from a spindle pole based on configuration in the physiological conditions (Kitamura et al., 2007) after setting parameters defining KT motion and MT dynamics in space and time (Fig. 5 A and Video 1; Gandhi et al., 2011). Most parameter values were obtained from previous live-cell imaging studies (see the Spatiotemporal simulation of KT–MT interaction section of Materials and methods; Kitamura et al., 2007, 2010; Tanaka et al., 2007; Brun, 2011; Gandhi et al., 2011; Kalinina et al., 2013). In this spatiotemporal simulation, we evaluated the efficiency of KT capture by quantifying the decrease of noncaptured KTs.

Using the spatiotemporal simulation, we first recapitulated the effect of KT-derived MTs on KT capture by spindle MTs. Because multiple short MTs extend and shrink repeatedly in all directions from KTs (Kitamura et al., 2010), for simplicity, we modeled KTs with a larger capture radius when KT-derived MTs were present (Fig. 5 A). If a spindle MT is present within the KT capture radius, a KT–MT interaction is formed. KTs always have a shorter capture radius in the absence of KT-derived MTs (Stu1-depleted cells), whereas KTs switch stochastically between a short and a longer capture radius in *STU1* wild-type cells. In wild-type cells, the frequency and duration of a longer capture radius were set to match those of KT-derived MTs observed in live-cell imaging (Fig. S3 A, Stu1 wild-type). Based on this modeling, we first determined a shorter KT capture radius (200 nm) to recapitulate the decline curve of noncaptured KTs in Stu1-depleted cells (Fig. 5 B, red; and Fig. S5 A). We then determined a longer KT capture radius (500 nm) to recapitulate the decline curve of noncaptured KTs with the shorter radius of 200 nm in *STU1* wild-type cells (Fig. 5 B, black; and Fig. S5, B–D). The values (500 and 200 nm) for a longer and shorter KT capture radius are consistent with the typical length of a KT-derived MT (Kitamura et al., 2010) and the size of purified KTs (Gonen et al., 2012), respectively. We therefore used these parameter values in the following analyses.

Next, we addressed how KT-derived MTs, KT diffusion, and MT pivoting facilitate KT capture by spindle MTs (Fig. 5 A). We switched off each of these factors (Videos 2, 3, and 4) or two in combination. For example, to switch off KT-derived MTs, the KT capture radius was set to 200 nm all the time. To switch off KT diffusion, we stopped the diffusional motion of KTs upon their reassembly. We then investigated how the fraction of noncaptured KTs declined along time (Fig. 5, C and D). The decline largely followed a simple exponential decay curve (Fig. S5, E–G), and we determined the half-life of noncaptured KTs in each condition. Intriguingly, when either KT diffusion or KT-derived MTs were absent, the half-lives of noncaptured KTs were longer by 7.6 and 17.5 s, respectively, compared with the wild type (Fig. 5 E, blue and orange bars). However, when MT pivoting was absent, the half-life showed little change (Fig. 5 E, green bar). Next, we analyzed the half-lives of noncaptured KTs when two factors were absent in combination (Fig. 5 E, magenta bars). If the change in a half-life (relative to that in a wild type) in the absence of two factors was greater than the sum of changes in the absence each factor (additive effect), we defined it as a synergistic effect. KT-derived MTs and KT diffusion showed the largest synergistic effect (+11.2 s; relative to an additive effect), whereas KT-derived MTs and MT pivoting also showed some synergistic effect (+5.9 s). Meanwhile, KT diffusion and MT pivoting showed a small synergistic effect (+2.3 s). Thus, MT pivoting is redundant with the other two factors in facilitating KT capture, as its absence alone changes a half-life very little, but it has synergistic effects when combined with them. Crucially, these results highlight two central roles of KT-derived MTs in facilitating KT capture by spindle MTs: (1) their effect on their own is the largest among the three factors studied here, and (2) it synergistically enhances the effect of KT diffusion and MT pivoting.

## Discussion

There are three major implications of this study. First, we have shown molecular mechanisms for generating MTs at KTs in budding yeast. Our previous study showed that Stu2 plays a central role in generating MTs with distal plus ends at KTs (Kitamura et al., 2010). We show here that Stu1 is required to recruit Stu2 to KTs and thereby to promote MT generation at KTs. Stu1 and Stu2 belong to the evolutionarily conserved CLASP and XMAP215/ch-TOG family, respectively, of MT-associated proteins (Al-Bassam and Chang, 2011). The following evidence suggests mechanisms similar to what we found in yeast may also promote MT generation at KTs in animal cells: (a) TACC3, a binding partner of ch-TOG (Peset and Vernos, 2008), associates with KTs to form MT asters in human cells (Fu et al., 2013); (b) CLASP is recruited to the KT before interaction with spindle MTs in human cells (Maffini et al., 2009); and (c) Stu1 and Stu2 orthologues physically interact to regulate MT dynamics in *Drosophila melanogaster* (Long et al., 2013). Meanwhile, it has been recently suggested that Stu2 at KTs is involved in error correction for biorientation based on in vitro observations (Miller et al., 2016). Unfortunately, we could not use Stu1 depletion (which reduces Stu2 at KTs) to address this issue in vivo because Stu1 depletion leads to a bipolar spindle defect (Yin et al., 2002), resulting in no biorientation.

Second, we have suggested that KT-derived MTs could facilitate KT capture by spindle MTs and quantified this effect; i.e., KT-derived MTs interact with spindle MTs and assist KT interaction with spindle MTs, shortening the half-life of noncaptured KTs from 48–49 s to 28–34 s (Fig. 3, B and D; and Fig. S3 D). Previous results showed correlation between the appearance of KT-derived MTs and rapid KT capture by spindle MTs (Kitamura et al., 2010; Fu et al., 2013), but more information was required to address whether the two are causatively related. In the current study, we found that Stu1 depletion led to both a lack of KT-derived MTs and a delay of KT capture by spindle MTs. Upon Stu1 depletion, it seemed that MT generation was abolished specifically at KTs without other significant changes in MT dynamics or KT assembly. We reason that a lack of KT-derived MTs in Stu1-depleted cells led to a delay in KT capture by spindle MTs, which is also supported by computational reconstitution of KT-derived MTs.

Third, using spatiotemporal computer simulation, we addressed how multiple factors (KT-derived MTs, KT diffusion, and MT pivoting) were involved independently and cooperatively in facilitating KT capture by spindle MTs. We identified redundancy and synergy in the effects of different factors. KT-derived MTs play central roles in KT capture because (a) their effect is greater than those of KT diffusion and MT pivoting, and (b) they synergistically enhance the effects of KT diffusion and MT pivoting. The initial KT encounter with a spindle MT is one of the limiting steps in establishing proper KT–MT interaction during mitosis, and further analyses are required to understand more about how this important process is regulated by multiple factors.

## Materials and methods

### Yeast strains and cell culture

The yeast strains used in this study were derivatives of W303 (K699 and K700 from K. Nasmyth, University of Oxford, Oxford, UK). The genotypes of strains used in this study are shown in Table S2. To synchronize cells in the cell cycle, yeast cells were arrested in G1 phase by treatment with yeast mating pheromone (α or a-factor) and subsequently were released to fresh media (Amberg et al., 2005; O’Reilly et al., 2012). The a-factor was produced by solid-phase peptide synthesis followed by two solution-phase steps for methylation of the carboxy terminal and farnesylation of the cysteine side chain (O’Reilly et al., 2012). Cells were cultured at 25°C in YPA (yeast extract, peptone, and adenine) medium containing 2% glucose (YPAD; Amberg et al., 2005) unless otherwise stated. To activate the *GAL* promoter, cells were preincubated in medium containing 2% raffinose (with 0.02% glucose) for at least for 3.5 h and subsequently were incubated in medium containing both 2% galactose and 2% raffinose. Cells were incubated in medium containing 2% glucose to suppress the *GAL* promoter without subsequent activation. The *MET3* promoter was activated by incubation of cells in methionine dropout media and was suppressed by adding 2 mM methionine to the relevant media. Constructs of *CEN5-tetOs* (Tanaka et al., 2000), *P_GAL_-CEN3-tetOs* (Hill and Bloom, 1987; Michaelis et al., 1997; Tanaka et al., 2005), *TetR-3×CFP* (Michaelis et al., 1997; Bressan et al., 2004), *P_MET3_-CDC20* (Uhlmann et al., 2000), *GFP-TUB1* (Straight et al., 1997), *CFP-TUB1* (Janke et al., 2002), *mCherry-TUB1* (Gandhi et al., 2011), *P_GALS_-STU2-GFP-LacI, STU2-GFP* (Kitamura et al., 2010), and *lacO*s on chromosome XII (Straight et al., 1996; Sullivan et al., 2004) were generated by us or by other groups with molecular cloning techniques using bacteria *Escherichia coli*. *P_GALS_-STU1-GFP-LacI* was constructed similarly to *P_GALS_-STU2-GFP-LacI.* The *STU1*, *STU2*, *NDC80*, *SPC105*, and *MTW1* genes were tagged with *3×GFP*, *4×mCherry*, and *3×CFP* at their C termini at their original gene loci by a one-step PCR method (Knop et al., 1999) using *3×GFP-KanMX6* (pSM1023; a gift from E. Schiebel, University of Heidelberg, Heidelberg, Germany; Maekawa et al., 2003), *4×mCherry-NatMX6* (pT909), and *3×CFP-HIS3* cassette (pT769; Gandhi et al., 2011) as PCR templates.

### Live-cell imaging and image analyses

During time-lapse imaging, yeast cells were immobilized on a glass-bottom dish (P35G-1.5-10-C; MatTek) coated with concanavalin A (C7275; Sigma-Aldrich), and maintained in either synthetic complete (SC) medium (Figs. 1, 2, S1, S2, and S4 G) or SC plus YPA medium (3:1 ratio; Figs. 3, 4, S3, and S4, A–C; Amberg et al., 2005; Tanaka et al., 2010). Images were acquired using a DeltaVision Core or Elite microscope (Applied Precision Ltd.), a UPlan S Apochromat 100× 1.40 NA objective lens (Olympus), SoftWoRx software (Applied Precision Ltd.), and a CoolSnap HQ camera (Photometrics). We acquired typically seven (0.7 µm apart) z sections, which were subsequently processed through deconvolution and analyzed with Volocity software (PerkinElmer). CFP, GFP, and mCherry signals were discriminated using a multiband filter set (89006; Chroma Technology Corp). For the image panels in figures, z sections were projected to 2D images.

### Centromere reactivation assay

To analyze individual KT–MT interactions in detail, a centromere reactivation assay was used (Tanaka et al., 2005, 2010). In this assay, KT assembly was delayed on a chosen centromere (*CEN3-tetOs* replacing *CEN3* on chromosome III or *CEN15* on chromosome XV) by transcription from the *GAL* promoter. This increased the distance between the centromere and the mitotic spindle, allowing detailed observation of KT–MT interactions after inducing KT assembly on the centromere by turning off the *GAL* promoter in metaphase-arrested cells (Fig. 1, A–D). Cells with *P_GAL_-CEN3-tetOs P_MET3_-CDC20* (see full genotypes in Table S2) were cultured overnight in methionine dropout media with raffinose, treated with mating hormone for 2.5 h (to arrest in G1 phase), and released to fresh media with raffinose, galactose, and 2 mM methionine (for Cdc20 depletion and *P_GAL_-CEN* inactivation). After 3.5–4 h, cells were suspended in SC medium containing glucose and methionine to reactivate *P_GAL_-CEN*. To define a contour of the spindle in Fig. S4 A, surface rendering was applied to the tubulin signals in z stacks using Imaris software (version 7; Bitplane).

### Analysis of KT capture by spindle MTs in physiological conditions

Individual KT signals were observed from their first appearance until capture by spindle MTs (Fig. S3 A). We reasoned that some events of KT reassembly and subsequent interaction with spindle pole MTs might not have been recognized when the time interval between the two was very short. This issue was addressed by obtaining a regression curve defined by f(*t*) = A* exp(−k*t), where A (scaling factor) and k are constant and *t* represents time (s), for the data (percentage of noncaptured KTs along time). With optimum A and k, we obtained a regression curve that intersects the y axis at f(0) = A. For plotting, both the regression curve and data (percentage) were divided by A for rescaling. After rescaling, a regression curve intersected the y axis at *t*(0) = 1. A and k values were as follows: A = 1.075, k = 0.021 (Fig. 3 B, black); A = 1.045, k = 0.014 (Fig. 3 D, magenta); A = 1.179, k = 0.025 (Fig. S3 D, black); and A = 0.997, k = 0.014 (Fig. S3 D, magenta). Meanwhile, to judge the KT capture, an overlap of a KT signal with an MT signal was not sufficient, as it may not have reflected a physical interaction of a KT with an MT because of the limit of resolution of light microscopy. So, we judged a KT capture only when a KT subsequently moved toward a spindle pole along the interacting MT. We defined KT capture timing as the time point before the detection of KT motion toward a pole where the KT signal and MT signal overlapped. Note that in a previous study (Kitamura et al., 2010), we defined KT capture timing when we detected the KT motion toward a pole, which was a slightly different definition from the current study. Therefore, the half-life of noncaptured KTs in Stu1 wild-type cells was shorter in the current study than in the previous study.

### Depletion of Stu1 and Stu2

To deplete Stu1 and Stu2 in the centromere reactivation assay, *STU1* and *STU2* were tagged with an *aid* tag at their C termini at original loci in the strain carrying the rice F-box gene *TIR1* (Nishimura et al., 2009). In the presence of synthetic auxin 1-naphthaleneacetic acid (NAA; 0.5 mM), *aid*-tagged proteins bound Tir1, which led to their ubiquitylation and degradation. To deplete Stu1 protein for short-interval imaging (5–10 s), an anchor away system (denoted as *stu1–anchor-away* in figure legends) was used (Haruki et al., 2008), which consisted of *STU1-FRB* (C-terminal tag at the original *STU1* locus), *RPL13A-2×FKBP12*, *TOR1-1*, and *fpr1Δ*. In the presence of rapamycin (10 µM), Stu1 protein bound Rpl13A ribosomal protein because of the FRB–FKBP12 interaction, which led to depletion of Stu1 in the nucleus. When *stu1–anchor-away* was used, control *STU1* + wild-type cells also carried *RPL13A-2×FKBP12*, *TOR1-1*, and *fpr1Δ* and were treated with rapamycin. We used *stu1–anchor-away* in these experiments instead of *stu1-aid* because NAA treatment tends to somewhat accelerate photobleaching of fluorescent proteins with short excitation intervals.

### Bimolecular fluorescence complementation assay

To address whether Stu1 and Stu2 show close association at KTs, *STU2* and *STU1* were tagged at their C termini at their original gene loci using *VN-TRP1* and *VC-HIS3MX3* cassettes containing N-terminal and C-terminal halves of the gene for Venus fluorescent protein (Sung and Huh, 2007). In cells with Stu1-VC and Stu2-VN, Venus signals were detected at clustered noncaptured KTs (Fig. 1 D), which is indicative of close association between Stu1 and Stu2 at KTs. A KT component, *MIF2*, was also tagged with VN and VC at its C terminus in the same way and was used as a control (Fig. S1 G). Note that (a) VC and VN do not cause the protein–protein association by themselves, and (b) VC and VN can rapidly form fluorescent Venus proteins in seconds once they are brought closely together (Gandhi et al., 2011). Also note that in nocodazole-treated cells, we identified the location of a spindle pole by the remaining Stu2 signal or a tubulin signal. KT signals not in the vicinity of a spindle pole were defined as clustered noncaptured KTs.

### Generation of KT-derived MTs by computational reconstitution

Using stochastic computational reconstitution, we added KT-derived MTs to the profile of Stu1-depleted cells as explained in Fig. S4 D. The number of events in Stu1-depleted cells was rescaled to the number of Stu1 wild-type cells, as explained in the Fig. S4 D legend. The addition of KT-derived MTs was based on the following properties of KT-derived MTs, as observed in Stu1 wild-type cells: (a) frequency of their appearance (the same number of KT-derived MTs as observed in Stu1 wild-type), (b) their duration (e.g., circles in Fig. S5 C), and (c) their success rate in assisting KT capture by a spindle MT. Meanwhile, in Figs. 4 E and S4 F, we computationally reconstituted KT-derived MTs in the same way but in groups of cells where half-lives of noncaptured KTs were scaled variously relative to the half-life in Stu1-depleted cells. Such a group of cells was obtained by rescaling of the events in Stu1-depleted cells to the number of events in Stu1 wild-type cells (see the Fig. S4 D legend) and then by scaling a lifetime of each event by a given factor (e.g., 1.5× for a 150% half-life and then rounded to the nearest integer).

### Statistical analyses

Statistical analyses were performed with Prism (GraphPad Software) by choosing an unpaired *t* test (Fig. 1, A–C; Fig. 2 C; Fig. 4, A–C; Fig. S1, D and F; Fig. S2 A; and Fig. S4, A–C), a log-rank test (Fig. 3, B and D; and Fig. S3 D), and Fisher’s exact test (Figs. 1 D, S1 G, and S4 G). The null hypotheses in these tests were that the samples were collected randomly and independently from the same population. All p-values were two-tailed, and the null hypotheses were reasonably discarded when p-values were <0.05. In *t* tests, data distribution was assumed to be normal, but this was not formally tested.

### Spatiotemporal simulation of the KT–MT interaction

We created a computer model and performed simulations of the initial KT–MT interaction (Figs. 5 and S5) based on configuration in the physiological conditions (Kitamura et al., 2007); i.e., MTs extend from a single spindle pole, capture KTs, and bring them back to the vicinity of the spindle pole. The simulation was previously developed (Gandhi et al., 2011), but several modifications were introduced in this study to more closely represent events in live cells. The values of the majority of the parameters were defined in previous studies of live-cell imaging and electron tomography (Tanaka et al., 2005, 2007; Kitamura et al., 2007, 2010; Brun, 2011; Gandhi et al., 2011; Kalinina et al., 2013; Natsume et al., 2013), and unknown parameters were measured in the current study. The parameter values used in this simulation are shown in Table S1. Whenever possible, the parameter values were estimated in physiological experimental conditions (Fig. 3; Kitamura et al., 2007). Only when it was difficult to estimate them in physiological conditions did we use an engineered condition (Fig. 1, A–D).

The model was computed as a discrete simulation of a series of events on a constant time step Δ*t*. All objects (MTs, KTs, and Stu2) were located in a 3D space in a Cartesian reference frame with the z axis pointing “up.” The nucleus was represented by a sphere of radius *R*_nuc_ (Natsume et al., 2013) centered along the z axis at the distance of *R*_nuc_ from the origin. A spindle pole was located at the origin. An exclusion radius, *r*_ex_, was established around the spindle pole. Each MT was a line segment extending into the nucleus from the spindle pole. Each KT was a point inside the nucleus. Stu2 was an MT polymerase that caused MT rescue (Gandhi et al., 2011) and was represented as a point.

MTs could grow and shrink with speed *v*_gro_ and *v*_shr_, respectively. A catastrophe (conversion from growth to shrinkage) could happen randomly at a rate of *K*_cat_ calculated only over the growth stage. When a growing MT hit the nuclear envelope, it started to shrink. When an empty MT shrank to r_ex_, it could start growing at a certain nucleation rate *K*_nuc_ unless there were KTs waiting at *r*_ex_, in which case the MT captured the KT and showed no further change. The value *K*_nuc_ was determined so that the 5–10 long MTs (>0.7 µm) appeared during KT collection, as estimated from electron tomography images in early S phase (e.g., Kitamura et al., 2007). When Stu2 moving along an MT reached its distal end, a shrinking MT was rescued; i.e., shrinkage was converted into growth (Gandhi et al., 2011). When the distal end of a shrinking MT caught up with a laterally sliding KT, it could be rescued with probability *P*_res_ (Gandhi et al., 2011). Otherwise, end-on pulling was commenced, and MTs were not rescued during end-on pulling (Tanaka et al., 2007).

MT direction was represented by two angles: the azimuth angle ϕ and the zenith angle θ (which was measured from the z axis). MTs grew and regrew in random directions with angular distribution concentrated toward the center of the nucleus. To simulate this situation, we initially generated an isotropic direction, ϕ = R[0, 2π] and cosθ = R[0, 1], where R[*a*, *b*] was a random number uniformly distributed between *a* and *b*. We transformed the zenith angle with a beaming factor, β:

$$\cos\theta^{\prime }=\frac{\cos\theta +\beta }{1+\beta \cos\theta }.$$

The angles ϕ and θ’ give the desired distribution, which is isotropic for β = 0 and becomes increasingly more concentrated toward the center of the nucleus as β approached 1. The value for β was estimated so that MTs extending from a spindle pole formed a configuration to images of electron tomography acquired in early S phase (e.g., Kitamura et al., 2007). The MTs also experienced “pivoting,” which was modeled by angular random walk with the diffusion coefficient *D*_MT_ (Brun, 2011; Kalinina et al., 2013).

Time 0 was defined as the mean time of replication of the first *CEN* (*CEN2*), and therefore its mean detachment time from a spindle pole. At −10 min, *n*_MT_ MTs started growing from a spindle pole (this number was set so that 5–10 long MTs [> 0.7 µm] appeared during KT collection). 16 MTs of length *r*_ex_ with attached centromeres were created at the start of the simulation. Each of these tied MTs became available for growth and shrinkage when replication of each centromere (Yabuki et al., 2002) caused KT disassembly and centromere detachment from the MT. Mean replication timing of each *CEN* is shown by Gandhi et al. (2011) based on the data by Yabuki et al. (2002). Variation was introduced in each *CEN* replication/*CEN* detachment timing by adding a random number *N*[0, *S^2^*_tim_]. After detachment, a centromere could move freely by a random walk with a diffusion coefficient *D*. After a delay *t*_del_, a KT was reassembled at the centromere’s position.

KTs moved freely inside the nucleus (except for the volume within the exclusion radius) after a random walk with a diffusion coefficient *D*. Once attached to an MT, a KT slid laterally toward the spindle pole or was pulled by the distal end of the MT with speed *v*_lat_ or *v*_pul_, respectively. The sliding motion was varied by a linear diffusion with a coefficient *D*_lat_. When a sliding KT reached the exclusion radius, it remained there until an empty MT shrank to *r*_ex_. Then the KT was caught at the end of this MT, and the “arrival” of this KT to the spindle pole was completed, after which no further change occurred to this KT and MT apart from MT pivoting. The same happened immediately when an end-on pulled KT reached the spindle pole.

A KT, which was sliding on an MT or waiting for end-on establishment at the spindle pole, could send Stu2 along the MT at a rate of *K*_stu2_ (Gandhi et al., 2011). Stu2 moved along the MT away from a spindle pole at a speed of *v*_stu2_. The arrival of Stu2 at the MT plus end led to rescue of the MT (rescue distal to the KT). When the MT plus end caught up with a KT, which was associated on the lateral side of this MT, MT rescue (rescue at the KT) occurred with the probability of *P*_res_ after a delay period of *t*_d_, during which the KT was pulled by slow-speed end-on pulling (speed *v*_spul_; discussed as MT pausing by Gandhi et al., 2011). The duration of the delay was estimated based on the duration of transient colocalization of the Dam1 complex with *CEN* in physiological conditions (Kitamura et al., 2007).

The interaction between KT-generated and spindle-pole MTs was simplified by assuming a certain capture radius, *R*_KT_, around each KT. If a KT was found at a distance *R*_KT_ from any part of a spindle-pole MT, the KT-derived MT connected to this spindle-pole MT by the shortest distance and brought the KT toward the spindle-pole MT, usually on its lateral side, at a speed *v*_cap_, which we assumed to be the same as the depolymerization rate of a KT-derived MT (Kitamura et al., 2010). Once capture was completed, the KT began sliding, which was converted to end-on pulling if end-on attachment was subsequently established.

Capture radius switched randomly between two values: “short,” R*_KT_* = *R*_short_; and “long,” R*_KT_* = *R*_long_. The switch from *R*_short_ to *R*_long_ occurred randomly at a rate *K*_long_. Once in the “long” state, capture radius could randomly switch to the short rate according to an exponential decay law, Pr(*t*) = 1 − exp(−*t*/τ_long_), where Pr(*t*) is the probability of the long-to-short switch at time *t* after the short-to-long switch. τ_long_ was established to reproduce the observed distribution of the duration of KT-derived MTs (Fig. S5 C, circles).

If an end-on pulled KT caught up with another KT that was sliding along the same MT, the sliding KT detached. We assumed that the detached KT was not able to reattach to an MT until the KT generated an MT from it at its maximum length *R*_KT_; i.e., for *R*_KT_/*v*_gro_ min (note that KT-derived MTs showed a similar growth rate to spindle-pole MTs; Kitamura et al., 2010).

The code for the simulation was written in Perl, and simulations were run in a Linux environment. To analyze the decline of noncaptured KTs, we ran 100,000 individual simulations in each condition. During the time window of 2–10 min, we analyzed *CEN*s that reassembled the KT after detachment from a spindle pole as a result of *CEN* replication and were captured by spindle MTs to mimic the observation in live-cell imaging in Figs. 3 and S3 A. Based on this dataset, we calculated the curve of the decline of noncaptured KTs in Fig. 5 B. To address how KT-derived MTs, KT diffusion, and MT pivoting facilitate KT capture by spindle MTs, we switched off each of these factors or two in combination (Fig. 5, C and D). For example, to switch off KT-derived MTs, the KT capture radius was set to 200 nm all the time (Video 2; compare with Video 1). To switch off KT diffusion, we stopped the diffusional motion of KTs upon their reassembly (Video 3; compare with Video 1). Decline curves of noncaptured KTs represent the proportion of *n* = 1.6 × 10^6^ KTs (i.e., 16 × 100,000; yeast has 16 KTs) that are noncaptured by spindle MTs at a given time. So, the maximum standard error of the fraction of noncaptured KTs is the square root of 0.25/*n* (0.25 is the maximum value of *p* × (1 − *p*), where *p* is a fraction and <1), which is <0.001.

### Online supplemental material

Figs. S1, S2, S3, S4, and S5 show supplemental results associated with Figs. 1, 2, 3, 4, and 5, respectively. Fig. S1 illustrates five factors that could facilitate the KT capture by spindle MTs in a table and diagram (A); shows that Stu1 localized at KTs before they are captured by spindle MTs, and Stu1 signals appear at KTs before MT/tubulin signals appear there (B); demonstrates that cells with Stu1 and Stu2 tagged with an *aid* are unable to grow in the presence of auxin NAA (auxin-induced depletion; C; Nishimura et al., 2009); shows that Stu1 depletion leads to reduction of Stu2 signals at KTs (D); indicates that both Stu1 and Stu2 localize at KT clusters in nocodazole-treated cells (E); shows Stu1 is required for Stu2 localization at KT clusters in nocodazole-treated cells (F); and suggests that Stu1 and Stu2 are closely associated at noncaptured KTs (G). Fig. S2 suggests that the Stu2-GFP-LacI level at *lacO*s is often higher than the Stu2-GFP level at the KT, but even in cells where these levels are similar, MT/tubulin signals appear at the Stu2-GFP-LacI–tethered site after depletion of Stu1 (A); shows in a diagram that Stu1-GFP-LacI is tethered at *lacO*s on a chromosome arm locus (B); suggests that accumulation of Stu1-GFP-LacI at *lacO*s on a chromosome arm locus is insufficient for MT generation (C); and shows that Stu1-GFP-LacI is able to generate MTs at KTs when it is the sole source of Stu1 in cells (D). Fig. S3 shows individual events of KT appearance and capture by a spindle MT in Stu1 wild-type and Stu1-depleted cells (A); shows that, if we replot the decline curve of noncaptured KTs in Stu1 wild-type cells using randomly selected points as new time 0s, we obtain similar curves to the original decline curve (B); demonstrates that cells with Stu1 tagged with FRB and a ribosome protein tagged with FKBP are unable to grow in the presence of rapamycin (anchor-away depletion in the nucleus; C; Haruki et al., 2008); and shows that the results in Fig. 3 (B and D) are reproducible using an independently obtained second dataset (D). Fig. S4 suggests that the intensity of the spindle is similar between Stu1 wild-type and Stu1-depleted cells (A); suggests that there is no significant difference in the Ndc80 and Spc105 signal intensity at noncaptured KTs between Stu1 wild-type and Stu1-depleted cells (B and C); shows how KT-derived MTs were added to Stu1-depleted cells in computational reconstitution (D); suggests that computational reconstitution of KT-derived MTs in Stu1-depleted cells recapitulates the decline curve of noncaptured KTs observed in Stu1 wild-type cells in the second dataset obtained in Fig. S3 D (E); suggests that Stu1-depleted cells show a delay in KT capture whose extent falls within a relatively narrow range expected from a lack of KT-derived MTs in computational reconstitution of KT-derived MTs with the second dataset obtained in Fig. S3 D (F); and supports the notion that a spindle MT captures a KT when they come close to each other more efficiently in the presence of Stu1 than its absence. Fig. S5 highlights how parameter values were optimized in spatiotemporal simulation of KT interaction with spindle MTs to reproduce observation in live-cell imaging (A–C); shows a decline curve of noncaptured KTs with a fixed KT capture radius (400 nm), which was the parameter value used in a previous study (D; Gandhi et al., 2011); and indicates that the decline curves of noncaptured KTs in simulations are very similar to simple exponential decay curves in various conditions (E–G). Table S1 lists parameter values used in spatiotemporal simulation of KT–MT interactions. Table S2 shows the genotypes of yeast strains used in this study. Video 1 shows an example of spatiotemporal simulation of KT–MT interaction with wild-type conditions. Video 2 shows an example of a spatiotemporal simulation of KT–MT interaction in the absence of KT-derived MTs. Video 3 shows an example of a spatiotemporal simulation of KT–MT interaction in the absence of KT diffusion. Video 4 shows an example of a spatiotemporal simulation of KT–MT interaction in the absence of MT pivoting.

## Supplementary Material

Supplemental Materials (PDF)

Table S2 (Excel)

Video 1

Video 2

Video 3

Video 4
