# Supplementary material for: Molecular mechanisms facilitating the initial kinetochore encounter with spindle microtubules
Source: J Cell Biol. 2017 Jun 5;216(6):1609–22. doi: 10.1083/jcb.201608122 (PMC5461016; doi:10.1083/jcb.201608122)
Supplement: Supplemental Materials (PDF) [file JCB_201608122_sm.pdf]

Vasileva et al., https://doi.org/10.1083/jcb.201608122

THE JOURNAL OF CELL BIOLOGY

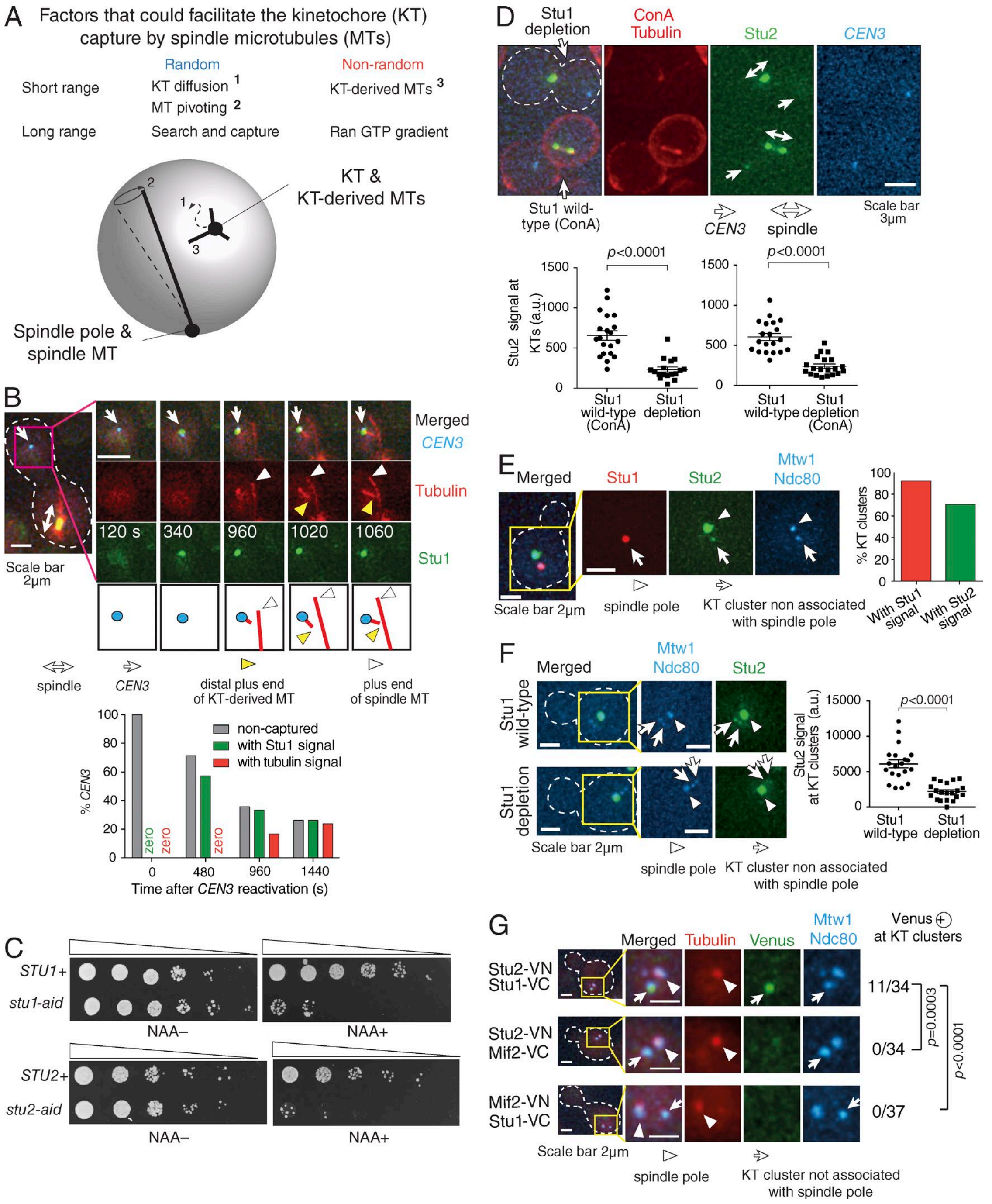

Figure S1. **Supplemental results associated with Fig. 1.** (A) The diagram illustrates factors that could facilitate the KT capture by spindle MTs. Five factors are classified as those promoting this process at a short range (up to 2–3 µm) and at a long range (>5 µm). They are also classified as working in random and nonrandom directions. The Ran-GTP gradient is a "nonrandom" factor because its concentration is higher around a chromosome and "guides" extension of spindle MTs toward it (Clarke and Zhang, 2008). KT-derived MTs extend in random directions from a KT (Kitamura et al., 2010), but only those that extend toward spindle MTs and interact with them are thought to be effective in facilitating the KT capture by spindle MTs. Based on this, KT-derived MTs are classified to a nonrandom factor. (B) Stu1 localized at KTs before they are captured by spindle MTs. Stu1 signals appear at KTs before MT/tubulin signals appear there. *STU1-4xmCherry* P_{GAL}*-CEN3-tetOs TetR-3xCFP GFP-TUB1* P_{MET3}*-CDC20* (T9778) cells were treated as in Fig. 1 A, except NAA was not added, and images were acquired every 20 s. An example of a cell is shown here. The bottom graph shows the percentage of *CEN3* (*n* = 42), which was not yet captured by spindle MTs (gray; defined as 100% at time 0) with Stu1 (green) and tubulin (red) signals. Note that Stu1 does not localize at the MT plus end here, and after its depletion, Stu2 is significantly reduced at noncaptured KTs but not at the MT plus end (Fig. 1 A). (C) Cells with Stu1 and Stu2 tagged with an *aid* are unable to grow in the presence of auxin NAA (auxin-induced depletion; Nishimura et al., 2009). *STU1*⁺ (T10699), *stu1-aid* (T10967), *STU2*⁺ (T10833), and *stu2-aid* (T10834) cells with *TIR1* were serially diluted by fivefold and incubated for 2 d with or without 0.5 mM auxin NAA. *Cont.*

(D) Stu1 depletion leads to reduction of Stu2 signals at KTs. We aimed to confirm the result in Fig. 1 A by observing wild-type Stu1 and Stu1-depleted cells in the same microscopy field. To discriminate one type of cells from the other, we stained the cell wall of one type of cells (but not the other) by CF594-labeled concanavalin A (ConA). We then mixed two types of cells together and observed them in the same microscopy field. *STU1*$^{+}$ (T10699) and *stu1-aid* (T10697) cells with *TIR1* P_{GAL}*-CEN3-tetOs TetR-3×CFP mCherry-TUB1 STU2-3×GFP* P_{MET3}*-CDC20* were treated as in Fig. 1 A and incubated in the presence and absence of 50 µg/ml concanavalin A for 20 min. After mixing the two types of cells, images were acquired as in Fig. 1 A. In the image shown here, Stu1 wild-type cells were stained by concanavalin A, whereas Stu1-depleted cells were not. Graphs show Stu2 signal intensity at noncaptured KTs when the Stu1 wild type was stained by concanavalin A (left; *n* = 20 and 17 for Stu1 wild type and Stu1 depletion, respectively) and when Stu1-depleted cells were stained by concanavalin A (right; *n* = 20 in each condition). (E) Both Stu1 and Stu2 localize at KT clusters in nocodazole-treated cells. *STU1-4×mCherry STU2-3×GFP MTW1-3×CFP NDC80-3×CFP CFP-TUB1* (T12669) cells were treated as in Fig. 1 D. Graph shows the percentage of KT clusters not locating at a spindle pole (*n* = 65), which are associated with Stu1 (red) and Stu2 (green) signals. (F) Stu1 is required for Stu2 localization at KT clusters in nocodazole-treated cells. *STU1*$^{+}$ (T12681) and *stu1-aid* (T12680) cells with *TIR1 STU2-3×GFP MTW1-3×CFP NDC80-3×CFP mCherry-TUB1* were treated as in Fig. 1 D, except that at 30 min before the release from a mating pheromone, auxin NAA was added to the media (NAA was also present after the release). Graph shows Stu2 signal intensity at noncaptured KTs in Stu1 wild type and Stu1 depletion. *n* = 20 in each condition. P-values (two tailed) were obtained by unpaired *t* tests. a.u., arbitrary unit. (G) Stu1 and Stu2 are closely associated at noncaptured KTs. The experiment shown in Fig. 1 D was repeated using Mif2-VN and Mif2-VC as negative controls. Mif2 is a KT component and the yeast orthologue of CENP-C in metazoan cells. *STU2-VN STU1-VC* (T12563), *STU2-VN MIF2-VC* (T12973), and *MIF2-VN STU1-VC* (T12974) cells with *mCherry-TUB1 MTW1-3×CFP NDC80-3×CFP* were treated, and images were acquired and analyzed as in Fig. 1 D. Numbers of bright KT clusters (not associated with a spindle pole; denominators) and those with Venus signals (numerators) are shown on the right. White dashed outlines show the shape of yeast cells. P-values (two tailed) were obtained by Fisher's exact test.

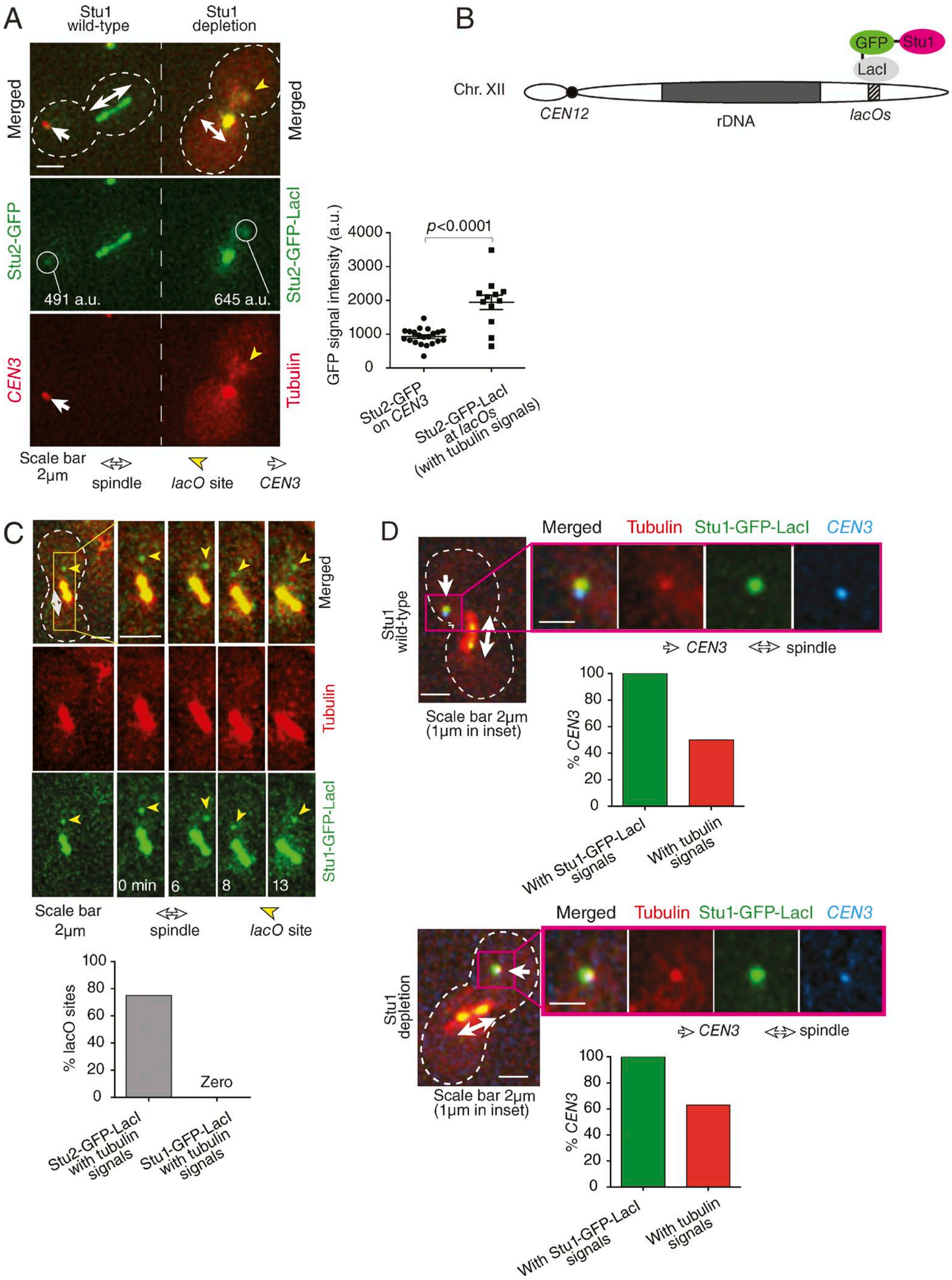

Figure S2. **Supplemental results associated with Fig. 2.** (A) The Stu2-GFP-LacI level at *lacO*s is often higher than the Stu2-GFP level at the KT. However, even in cells where these levels are similar, MT/tubulin signals appear at the Stu2-GFP-LacI–tethered site after depletion of Stu1. *STU1*$^+$ *STU2-GFP* P_{GAL}-*CEN3-tetOs TetR-3xCFP* P_{MET3}-*CDC20* (T5477) and *stu1-aid TIR1* P_{GAL}-*STU2-GFP-LacI REC102:lacO*s *CFP-TUB1* P_{MET3}-*CDC20* (T11686) cells were treated as in Figs. S1 B and 2 B, respectively. From 2 h after the release, galactose (to express Stu2-GFP-LacI) and auxin NAA (to deplete Stu1-aid) was added to the media. T5477 and T11686 cells were mixed and then suspended in media containing glucose to reactivate *CEN3* and to stop expression of Stu2-GFP-LacI. An example of the image is from the same microscopy field; two kinds of cells were distinguished by the presence/absence of CFP-Tub1 signals. Graph shows the GFP signal intensity at *CEN3-tetOs* or *REC102-lacO*s (n = 21 and 12 for the left and right, respectively). For the latter, Stu2-GFP-lacI at *lacO*s was associated with tubulin signals in ~80% cells (also see Fig. 2 B), and GFP signals were quantified in such cells. The p-value (two tailed) was obtained by an unpaired t test. a.u., arbitrary units. (B) Diagram showing that Stu1-GFP-LacI is tethered at *lacO*s on a chromosome arm locus. rDNA, ribosomal DNA. (C) Accumulation of Stu1-GFP-LacI at *lacO*s on a chromosome arm locus is insufficient for MT generation. P_{GALS}-*STU1-GFP-LacI REC102:lacO*s *CFP-TUB1* P_{MET3}-*CDC20* (T11106) cells were treated as in Fig. 2 B. Images were acquired every 1 min. Time 0 was set arbitrarily. The percentage of *lacO*s that are associated with MT/tubulin signals was compared between tethering of Stu2-GFP-LacI (n = 28; Fig. 2 B) and Stu1-GFP-LacI (n = 20). (D) Stu1-GFP-LacI is able to generate MTs at KTs when it is the sole source of Stu1 in cells. *STU1*$^+$ (expressed from *STU1* promoter) P_{GAL}-*STU1-GFP-LacI* P_{GAL}-*CEN3-tetOs TetR-3xCFP mCherry-TUB1* P_{MET3}-*CDC20* (T11263) cells (example is shown at top) were treated as in Fig. S1 B. *stu1-aid* (expressed from *STU1* promoter) P_{GAL}-*STU1-GFP-LacI TIR1* P_{GAL}-*CEN3-tetOs TetR-CFP* P_{MET3}-*CDC20 mCherry-TUB1* P_{MET3}-*CDC20* (T11339) cells (example is shown at bottom) were treated as in Fig. 1 A. Graphs show the percentage of *CEN3* (top, n = 36; bottom, n = 35) associated with Stu1-GFP-LacI (green) and tubulin (red) signals. The result suggests that a lack of MT/tubulin signals at the Stu1-GFP-LacI–tethered site in C was not caused by an impaired Stu1 function as a result of its fusion with GFP-LacI. White dashed outlines show the shape of yeast cells.

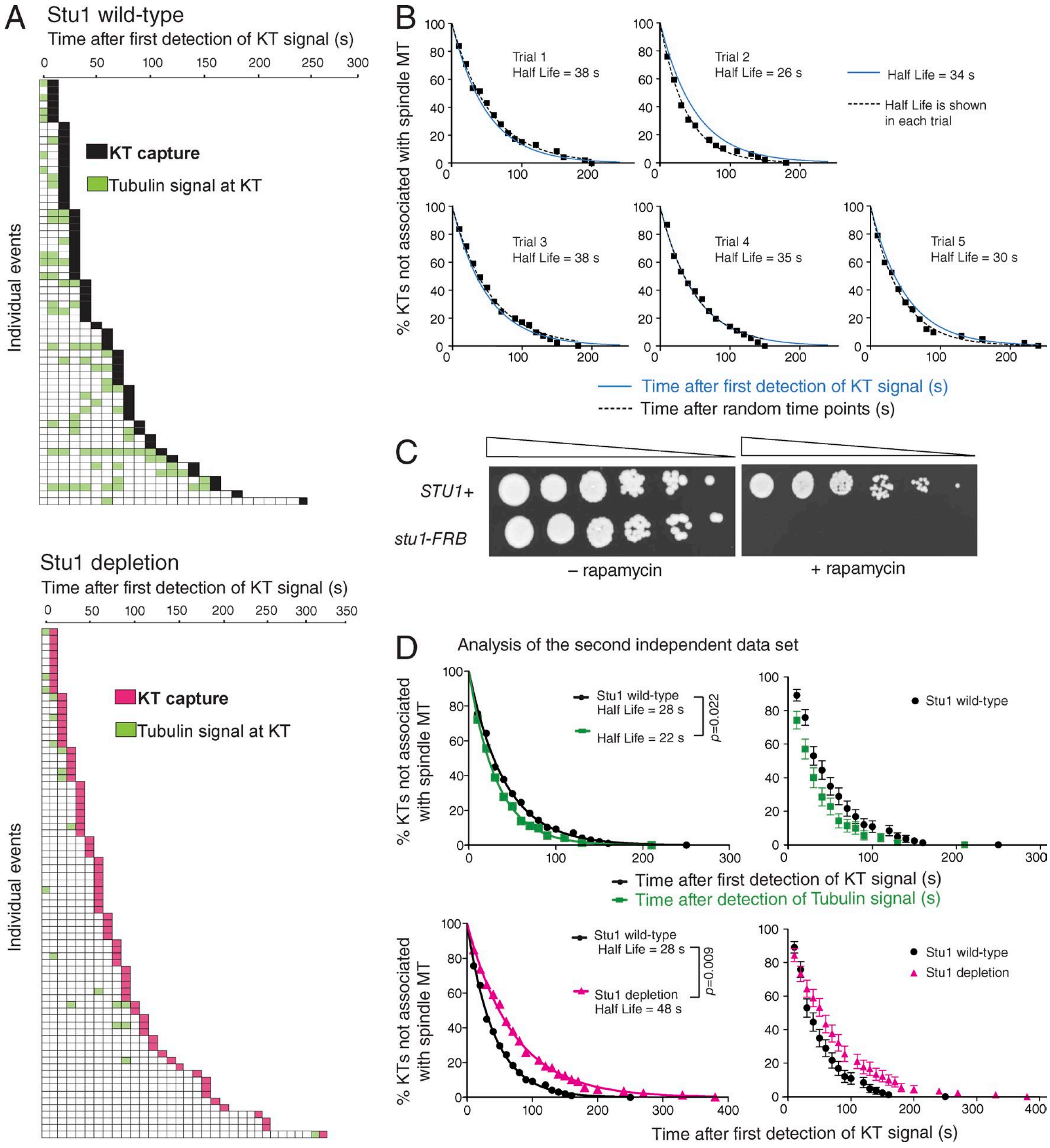

Figure S3. **Supplemental results associated with Fig. 3.** (A) An individual event of KT appearance and capture by a spindle MT is shown in each row in Stu1 wild-type (T11242, left; *n* = 60) and Stu1-depleted (T11230, right; *n* = 74) cells. Images were acquired every 10 s in physiological conditions, as explained in Fig. 3; each time point corresponds to each box shown here. A KT signal was first detected at time 0 and was followed until captured by a spindle MT (black box in left; magenta box in right). Timing of KT capture was determined when a KT signal overlapped with an MT signal followed by the KT motion toward a spindle pole. Tubulin signals were associated with noncaptured KTs at the time points highlighted in green. (B) If we replot the decline curve of noncaptured KTs using randomly selected points as new time 0, we obtain similar curves to the original decline curve. In A (Stu1 wild-type cells), from all the time points of all cells, we randomly chose one time point of one cell. For this, we numbered all these time points before KT capture and randomly picked one number. We repeated this process and chose the same number of time points as KT-associated tubulin signals (consecutive observation of tubulin signals at a KT was considered to be one signal). Then, we defined such randomly chosen time points as time 0 and drew a decline curve of noncaptured KTs, which was then fitted by a simple exponential decay curve. We repeated this process five times (Trials 1–5) to draw five decline curves of noncaptured KTs (dashed lines). Although there were some variations, dashed lines were overall similar to the original decline curve of noncaptured KTs (blue lines). (C) Cells with Stu1 tagged with FRB and a ribosome protein tagged with FKBP were unable to grow in the presence of rapamycin (anchor-away depletion in the nucleus; Haruki et al., 2008). *STU1+* (T11242) and *STU1-FRB* (*stu1–anchor-away*; T11230) cells with *TOR1-1 frp1Δ RPL13A-2×FKBP12* were serially diluted by fivefold and incubated for 2 d with and without 10 µM rapamycin. (D) Results in Fig. 3 (B and D) are reproducible. To test reproducibility of the results shown in Fig. 3 (B and D), we repeated experiments described in Figs. 3 and S3 A and obtained a dataset similar to Fig. S3 A but independent of it. This dataset contained 83 and 90 events of KT capture in Stu1 wild-type and Stu1-depleted cells, respectively. This dataset was analyzed in the same way as in Fig. 3 (B and D); i.e., the percentage of noncaptured KTs (100% at time 0; time 0 is the first time point when a noncaptured KT was detected) was plotted and fitted to a simple exponential decay curve in wild-type cells (top left and bottom left, black dots and line) and in Stu1-depleted cells (bottom left, magenta triangles and line). A KT-associated MT/tubulin signal appeared at a single or consecutive time points in Stu1 wild-type cells, and the first time point of each appearance was defined as new time 0 for replotting the percentage of noncaptured KTs (top left, green squares and line). Half-lives were calculated based on fitted exponential decay curves. Error bars represent a standard error of proportion for the data points (top right and bottom right). The p-value (two tailed) was obtained by a log-rank test for survival curves. These results suggest that (a) appearance of KT-derived MTs is correlated with more rapid KT capture by spindle MTs, and (b) Stu1 depletion leads to a delay in KT capture, both of which are consistent with our results in Fig. 3 (B and D).

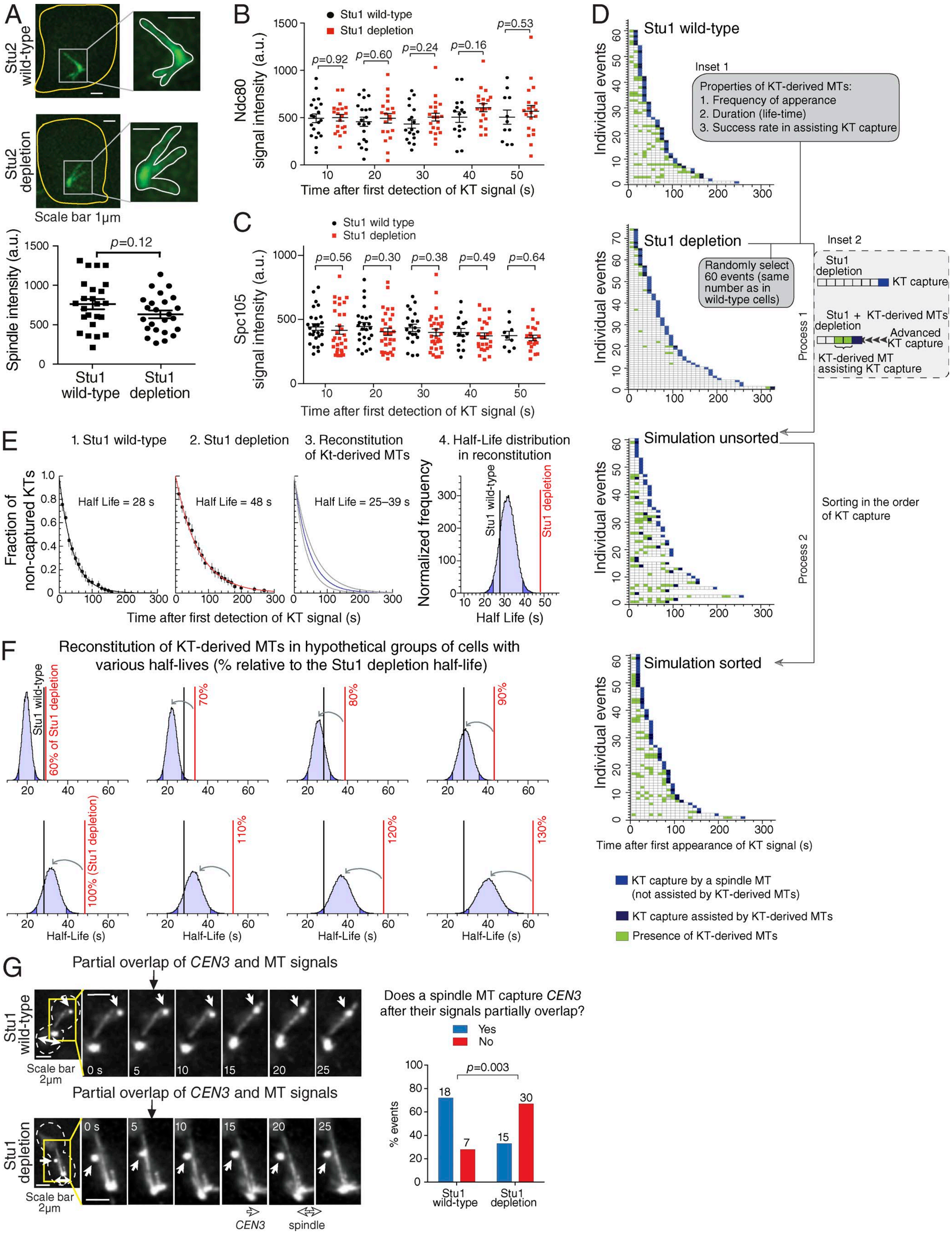

A
Stu2 wild-type
Stu2 depletion
Scale bar 1μm
Spindle intensity (a.u.)
p=0.12
Stu1 wild-type
Stu1 depletion
B
Stu1 wild-type
Stu1 depletion
Ndc80 signal intensity (a.u.)
p=0.92
p=0.60
p=0.24
p=0.16
p=0.53
Time after first detection of KT signal (s)
C
Stu1 wild type
Stu1 depletion
Spc105 signal intensity (a.u.)
p=0.56
p=0.30
p=0.38
p=0.49
p=0.64
Time after first detection of KT signal (s)
D
Stu1 wild-type
Individual events
Inset 1
Properties of KT-derived MTs:
1. Frequency of apperance
2. Duration (life-time)
3. Success rate in assisting KT capture
Stu1 depletion
Randomly select 60 events (same number as in wild-type cells)
Inset 2
Stu1 depletion
KT capture
Stu1 + depletion
KT-derived MTs
Advanced KT capture
KT-derived MT assisting KT capture
Process 1
Simulation unsorted
Sorting in the order of KT capture
Process 2
Simulation sorted
Time after first appearance of KT signal (s)
KT capture by a spindle MT (not assisted by KT-derived MTs)
KT capture assisted by KT-derived MTs
Presence of KT-derived MTs
E
1. Stu1 wild-type
2. Stu1 depletion
3. Reconstitution of Kt-derived MTs
4. Half-Life distribution in reconstitution
Fraction of non-captured KTs
Half Life = 28 s
Half Life = 48 s
Half Life = 25–39 s
Normalized frequency
Stu1 wild-type
Stu1 depletion
Time after first detection of KT signal (s)
Half Life (s)
F
Reconstitution of KT-derived MTs in hypothetical groups of cells with various half-lives (% relative to the Stu1 depletion half-life)
Stu1 wild-type
60% of Stu1 depletion
70%
80%
90%
100% (Stu1 depletion)
110%
120%
130%
Half-Life (s)
G
Partial overlap of CEN3 and MT signals
Stu1 wild-type
Scale bar 2μm
0 s
5
10
15
20
25
Stu1 depletion
CEN3
spindle
Does a spindle MT capture CEN3 after their signals partially overlap?
Yes
No
% events
p=0.003
18
7
15
30
Stu1 wild-type
Stu1 depletion

Figure S4. **Supplemental results associated with Fig. 4.** (A) Intensity of the spindle is similar between Stu1 wild-type and Stu1-depleted cells. Images obtained in Fig. 3 were analyzed further to quantify the total signal intensity of the spindle. Representative contours of the spindle are projected to 2D images (top). Signals of >50 per voxel in the default setting of the software were included in quantification. The total signal intensity of the spindle was quantified in individual cells (graph at bottom; *n* = 24 in each condition). (B) There is no significant difference in Ndc80 signal intensity at noncaptured KTs between Stu1 wild-type and Stu1-depleted cells. In Fig. 4 B, we quantified the intensity of Mtw1 and Ndc80 together to evaluate KT assembly. There was no significant difference in this intensity between Stu1 wild-type and Stu1-depleted cells. To address whether Stu1 depletion leads to a defect in the outer KT assembly, we next evaluated the intensity of only Ndc80. For this, *STU1*$^+$ (T12903) and *stu1–anchor-away* (T12901) cells with *NDC80-4×mCherry YFP-TUB1* cells were treated and analyzed as in Fig. 4 A. The intensity of the KT signal is plotted at each time point (time after the first appearance of the KT signal) in individual cells (*n* = 20 at each time point and in each condition, except for 30 s [*n* = 18], 40 s [*n* = 16], and 50 s [*n* = 11] in the Stu1 wild type). (C) There is no significant difference in Spc105 signal intensity at noncaptured KTs between Stu1 wild-type and Stu1-depleted cells. To investigate further whether Stu1 depletion leads to a defect in the outer KT assembly, we also evaluated the intensity of Spc105 at noncaptured KTs. Spc105 is an outer KT component and a yeast orthologue of KNL1. *STU1*$^+$ (T12988) and *stu1–anchor-away* (T12987) cells with *SPC105-4×mCherry YFP-TUB1* cells were treated and analyzed as in Fig. 4 A. The intensity of the KT signal is plotted at each time point (time after the first appearance of the KT signal) in individual cells (*n* = 27, 25, 21, 15, and 9 at 10–50 s, respectively, in the Stu1 wild type; *n* = 30, 30, 30, 25, and 21 at 10–50 s, respectively, in Stu1 depletion). The mean ± standard error is shown in each group of cells. P-values (two tailed) were obtained by unpaired *t* test. a.u., arbitrary unit. (D) KT-derived MTs were added to Stu1-depleted cells in computational reconstitution. Frequency of appearance of KT-derived MTs, their durations (lifetime), and their success rates in assisting KT capture were determined based on the data from Stu1 wild-type cells shown in Fig. S3 A, left (inset 1). For example, if KT capture immediately followed the presence of KT-derived MTs, we interpreted it as a successful assistance of KT capture by KT-derived MTs. Such properties of KT-derived MTs were applied to Stu1-depleted cells shown in Fig. S3 A (right) using stochastic computer simulation after the number of events in Stu1 depletion (74) was rescaled to the number of events in Stu1 wild-type cells (60; Process 1). This rescaling was done by randomly choosing 60 events from the events in Stu1 depletion without any overlap. In Process 1, if a "successful" KT-derived MT with a certain lifetime was assigned in a Stu1-depleted cell, we advanced the time point of KT capture accordingly (inset 2). Stu1-depleted cells originally showed tubulin signals at noncaptured KTs in a small number of time points; simulation added tubulin signals without considering these preexisting tubulin signals. The individual events, obtained by simulation, were sorted in the order of KT capture by spindle MTs (Process 2). The simulation was repeated 100,000 times and the integrated results are shown in Fig. 4 D. (E) Computational reconstitution of KT-derived MTs in Stu1-depleted cells recapitulates the decline curve of noncaptured KTs observed in Stu1 wild-type cells in the second dataset. In the second dataset (obtained in Fig. S3 D), KT-derived MTs were computationally added to Stu1-depleted cells, as in D. Results are presented in the same way as in Fig. 4 D. The half-life observed in *STU1* wild-type cells in vivo was within the 95% CI of half-lives of the reconstituted decline curves. (F) Stu1-depleted cells show a delay in KT capture whose extent falls within a relatively narrow range expected from a lack of KT-derived MTs in the computational reconstitution of KT-derived MTs. The second dataset (obtained in Fig. S3 D) was analyzed, and results are presented as in Fig. 4 E. (G) A spindle MT captures a KT when they come close to each other more efficiently in the presence of Stu1 than its absence. KT-derived MTs are relatively short in length (also see Figs. 1 B and 3 A; Kitamura et al., 2010). Therefore, if KT-derived MTs indeed facilitate KT capture by spindle MTs, this would happen only in close proximity to the spindle MTs. Therefore, in the presence and absence of KT-derived MTs, there would be a difference in the rate of KT capture by spindle MTs immediately after KTs come close to them. We tested this prediction using the centromere reactivation assay that allowed detailed analysis of KT interaction with a single spindle MT. *STU1*$^+$ (T11966) and *stu1–anchor-away* (T11965) cells with P_{GAL}-*CEN3-tetOs tetR-GFP GFP-TUB1* P_{MET3}-*CDC20* were treated as in Fig. 1 A, except rapamycin was added instead of auxin to deplete Stu1 in the nucleus. Images were acquired every 5 s. The top row shows *CEN3* capture by a spindle MT in a Stu1 wild-type cell. The bottom row shows the failure of *CEN3* capture in a Stu1-depleted cell after the partial overlap of a *CEN3* signal with a spindle MT signal (light microscopy resolution means that this overlap does not necessarily mean physical association). The graph shows the frequency of *CEN3* capture (blue) and no capture (red) after partial overlap between *CEN3* and a spindle MT. Capture was confirmed when *CEN3* subsequently moved along a spindle MT. Numbers on top of bars shows those of observed events. The p-value (two tailed) was obtained by Fisher's exact test. The result suggests that after the partial overlap between the centromere signal and a spindle MT signal, the centromere moved away from a spindle MT more often in the absence of KT-derived MTs (Stu1-depleted cells). The result is consistent with KT-derived MTs indeed facilitating KT capture by spindle MTs when KTs closely approach spindle MTs. White dashed outlines show the shape of yeast cells.

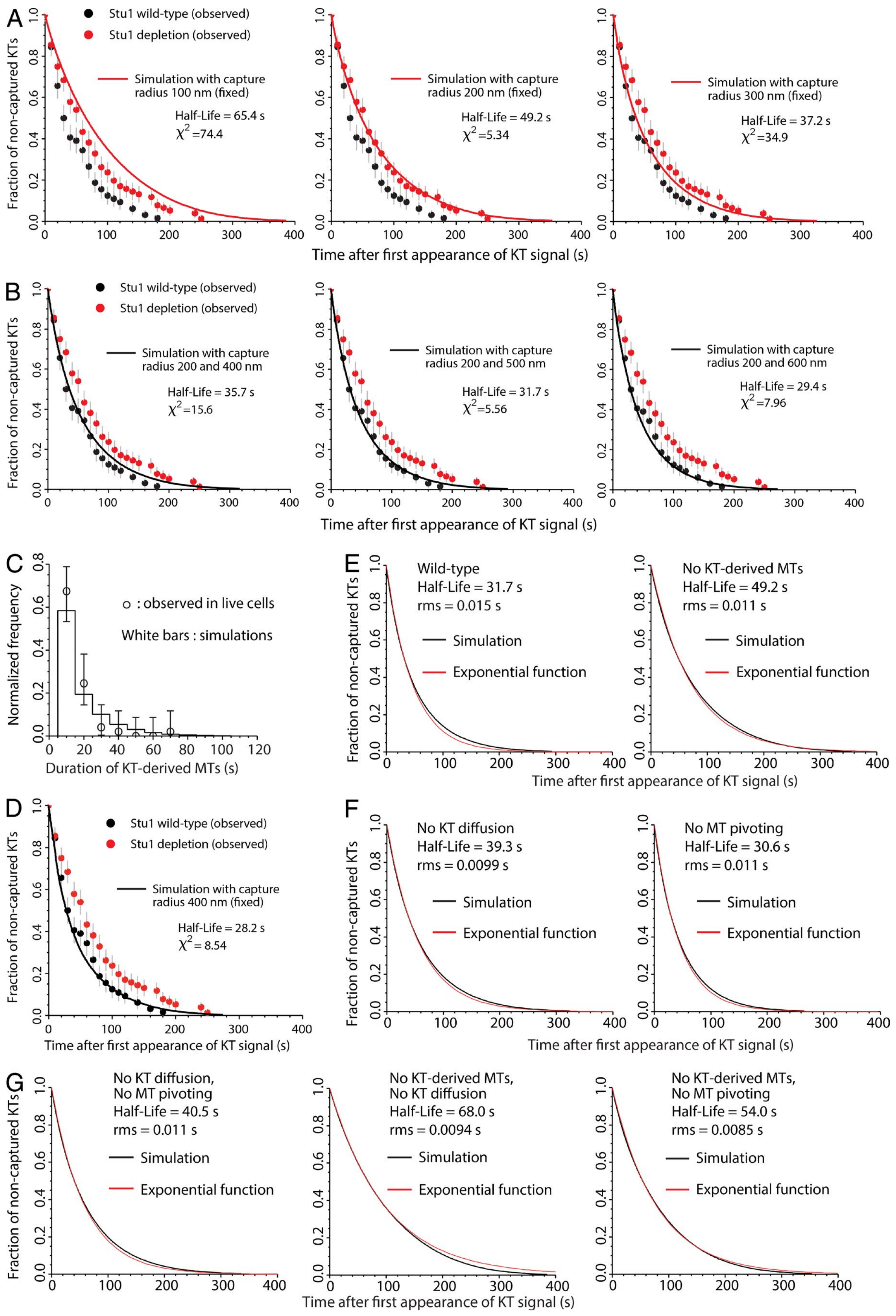
A
Stu1 wild-type (observed)
Stu1 depletion (observed)
Simulation with capture radius 100 nm (fixed)
Half-Life = 65.4 s
χ2 =74.4
Simulation with capture radius 200 nm (fixed)
Half-Life = 49.2 s
χ2 =5.34
Simulation with capture radius 300 nm (fixed)
Half-Life = 37.2 s
χ2 =34.9
Fraction of non-captured KTs
Time after first appearance of KT signal (s)
B
Stu1 wild-type (observed)
Stu1 depletion (observed)
Simulation with capture radius 200 and 400 nm
Half-Life = 35.7 s
χ2 =15.6
Simulation with capture radius 200 and 500 nm
Half-Life = 31.7 s
χ2 =5.56
Simulation with capture radius 200 and 600 nm
Half-Life = 29.4 s
χ2 =7.96
Fraction of non-captured KTs
Time after first appearance of KT signal (s)
C
○ : observed in live cells
White bars : simulations
Normalized frequency
Duration of KT-derived MTs (s)
E
Wild-type
Half-Life = 31.7 s
rms = 0.015 s
No KT-derived MTs
Half-Life = 49.2 s
rms = 0.011 s
Simulation
Exponential function
Fraction of non-captured KTs
Time after first appearance of KT signal (s)
D
Stu1 wild-type (observed)
Stu1 depletion (observed)
Simulation with capture radius 400 nm (fixed)
Half-Life = 28.2 s
χ2 = 8.54
Fraction of non-captured KTs
Time after first appearance of KT signal (s)
F
No KT diffusion
Half-Life = 39.3 s
rms = 0.0099 s
No MT pivoting
Half-Life = 30.6 s
rms = 0.011 s
Simulation
Exponential function
Fraction of non-captured KTs
Time after first appearance of KT signal (s)
G
No KT diffusion, No MT pivoting
Half-Life = 40.5 s
rms = 0.011 s
No KT-derived MTs, No KT diffusion
Half-Life = 68.0 s
rms = 0.0094 s
No KT-derived MTs, No MT pivoting
Half-Life = 54.0 s
rms = 0.0085 s
Simulation
Exponential function
Fraction of non-captured KTs
Time after first appearance of KT signal (s)

Figure S5. **Supplemental results associated with Fig. 5.** (A–C) Parameter values were optimized in spatiotemporal simulation to reproduce observation in live-cell imaging. We optimized parameter values defining (a) a KT capture radius (if a spindle MT is present within this radius, KT–MT interaction is formed) and (b) the original duration of KT-derived MTs (the original duration may be shortened if KT-derived MTs assist KT capture by spindle MTs). This optimization aimed to reproduce (a) the decline curves of noncaptured KTs with Stu1 wild-type (B) and Stu1 depletion (A) and (b) the distribution of duration of KT-derived MTs (C, white circles). Because multiple short MTs extend and shrink repeatedly in all directions from KTs (Kitamura et al., 2010), for simplicity, we modeled KTs with and without KT-derived MTs as follows: KTs always have a shorter capture radius in the absence of KT-derived MTs (Stu1-depleted cells), whereas KTs switch between a short and longer capture radius stochastically in wild-type cells. In wild-type cells, the frequency and duration of a longer capture radius were set to match those of KT-derived MTs observed in live-cell imaging. Based on this modeling, we first determined a shorter KT capture radius to recapitulate the decline curve of noncaptured KTs in Stu1-depleted cells. Panel A shows the decline curves with a KT capture radius of 100, 200, and 300 nm; 200 nm showed the best fit (smallest χ^2 value). We then determined a longer KT capture radius to recapitulate the decline curve of noncaptured KTs, using the shorter radius of 200 nm, in Stu1 wild-type cells. Panel B shows the decline curves with a longer KT capture radius 400, 500, and 600 nm; 500 nm showed the best fit. Note that in A and B, black and red circles show the observed fraction of noncaptured KTs along the time course in Stu1 wild-type and Stu1-depleted cells (with and without KT-derived MTs), respectively (also see Fig. 5 A; error bars are as in Fig. 4 D). Black and red lines show the results of spatiotemporal simulations (100,000× for each; this number of simulations results in typical half-life time standard errors of <0.3 s), and a half-life obtained in each condition is shown. χ^2, which represents the sum of $(x - y)^2/y$ (where x and y show the observed value and simulation outcome, respectively) shows the quality of simulation fitness to observed data in Stu1-depleted cells (A) and Stu1 wild-type cells (B); when χ^2 is smaller, fitness is better. Meanwhile, when switching between the short and long KTs, capture radius was defined by parameter values for K_{long} and τ_{long} as described in the Spatiotemporal simulation of KT–MT interaction section of Materials and methods, the distributions of duration of KT-derived MTs were similar to what was observed in live-cell imaging, as shown in C. In C, white circles show the frequency of duration of KT-derived MTs observed in live-cell imaging. Error bars show standard errors of proportions. White bars show the frequency of duration of KT-derived MTs obtained in simulation. (D) Modeling KT capture with a fixed KT capture radius of 400 nm. In the modeling of our previous study (Gandhi et al., 2011), we used a fixed KT capture radius of 400 nm. In this graph, the decline curve of noncaptured KTs is shown based on simulation with that condition (black line). The curve reasonably fits the observed data in Stu1 wild-type cells (χ^2 shows fitness to the observed data in Stu1 wild-type cells). However, the simulation with capture radii of 200 and 500 nm (corresponding to the absence and presence of KT-derived MTs, respectively) gives a better-fitting curve (a smaller χ^2 value; see B, middle) than does the simulation with the fixed capture radius 400 nm. (E–G) The decline curves of noncaptured KTs in simulations (black) are very similar to simple exponential decay curves (red) in various conditions. The red curve represents an exponential function, $\exp(-k*t)$, where *t* is time and k is a constant, with the same half-life (ln(2)/k) as in the corresponding black curve obtained in a spatiotemporal simulation (Fig. 5, B–D). rms, root mean square difference between red and black curves.

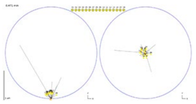

Video 1. **Example of spatiotemporal simulation of KT–MT interaction with wild-type conditions.** 3D simulations with wild-type conditions are projected into x/z and x/y planes. KTs locate in the vicinity of a spindle pole before centromere (*CEN*) DNA replication (yellow dots). Upon *CEN* replication, KTs disassemble, and *CEN*s move away from a pole (gray dots; Kitamura et al., 2007). KTs are then reassembled (red dots) on *CEN*s (when noncaptured KTs generate MTs, their diameters are enlarged), interact with the lateral side of MTs extended from a spindle pole (orange dots), and slide along an MT toward a spindle pole (green dots). KTs are then tethered at the MT end and transported polewards by MT end-on pulling (purple dots). Subsequently, they are tethered at the end of short MTs in the vicinity of the pole (blue dots). MT extension after KT-dependent rescue is shown as a dashed line (Gandhi et al., 2011). A gray line, which connects a KT (dot) to an MT extending from a spindle pole (black line), represents a KT-derived MT that facilitates KT loading onto an MT extending from a spindle pole (Kitamura et al., 2010). A KT capture radius (if a spindle MT is present within this radius, a KT–MT interaction is formed) is 500 and 200 nm in the presence and absence of KT-derived MTs, respectively. KTs with a 200- and 500-nm capture radius are symbolically represented by small and large dots, respectively; however, note that the radii of these dots do not correspond to the actual capture radius.

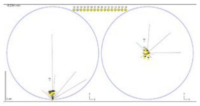

Video 2. **Example of spatiotemporal simulation of KT–MT interaction in the absence of KT-derived MTs.** 3D simulations in the absence of KT-derived MTs are projected into x/z and x/y planes. A KT capture radius remained 200 nm all the time. Otherwise, the simulation is shown as in Video 1.

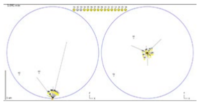

Video 3. **Example of spatiotemporal simulation of KT–MT interaction in the absence of KT diffusion.** 3D simulations in the absence of KT diffusion are projected into x/z and x/y planes. When a KT was assembled on a *CEN*, the KT motion stopped. Otherwise, the simulation is shown as in Video 1.

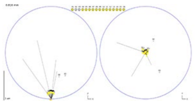

Video 4. **Example of spatiotemporal simulation of KT–MT interaction in the absence of MT pivoting.** 3D simulations in the absence of MT pivoting are projected into x/z and x/y planes. There is no angular diffusion (pivoting) of spindle MTs. Otherwise, the simulation is shown as in Video 1.

Table S1. **Parameter values used in spatiotemporal simulation of KT–MT interaction**

Parameter	Symbol	Value	Source of the value
Time step	Δt	0.001 min	A reasonably small value was chosen
Radius of the nucleus	R_{nuc}	1.25 µm	Natsume et al., 2013 (visualization of the nuclear envelope)
Initial MT number	n_{MT}	10	Based on Fig. S1 E, etc., in Kitamura et al., 2010
Exclusion radius	r_{ex}	0.2 µm	Based on Fig. S1 E, etc., in Kitamura et al., 2010
MT growth speed	v_{gro}	1.5 µm/min	Fig. 3 B in Tanaka et al., 2005
MT shrinkage speed	v_{shr}	2.8 µm/min	Fig. 3 B in Tanaka et al., 2005
MT catastrophe rate	K_{cat}	0.6 min^{-1}	Gandhi et al., 2011
MT nucleation rate	K_{nuc}	1 min^{-1}	Based on Fig. S1 E, etc., in Kitamura et al., 2010
MT beaming factor	β	0.7	Based on Fig. S1 E, etc., in Kitamura et al., 2010
MT angular diffusion coefficient	D_{MT}	0.03 rad^2/min	Based on Kalinina et al., 2013
Diffusion coefficient	D	0.1 µm^2/min	Fig. S1 in Kitamura et al., 2007
KT replication timing SD	S_{tim}	1 min	This study
KT activation delay	t_{del}	2 min	Gandhi et al., 2011
KT lateral sliding speed	v_{lat}	1 µm/min	Fig. 7 C in Kitamura et al., 2007
KT lateral sliding diffusion coefficient	D_{lat}	0.1 µm^2/min	Fig. 3 in Tanaka et al., 2007
KT end-on pulling speed	v_{pul}	1.7 µm/min	Fig. 2 D in Tanaka et al., 2007, and Fig. 7 C in Kitamura et al., 2007
KT slow end-on pulling speed	v_{spul}	0.35 µm/min	Gandhi et al., 2011
KT rescue delay	t_d	8 s	Gandhi et al., 2011
Stu2 sending rate	K_{stu2}	0.1 min^{-1}	Gandhi et al., 2011
Stu2 speed	v_{stu2}	2.1 µm/min	Fig. S9 in Tanaka et al., 2005; Gandhi et al., 2011
KT capture radius short	R_{short}	0.2 µm	This study
KT capture radius long	R_{long}	0.5 µm	This study
KT long capture radius rate	K_{long}	1 min^{-1}	This study
KT long capture radius decay coefficient	τ_{long}	0.5 min	This study
KT capture speed	v_{cap}	5 µm/min	Fig. S1 C in Kitamura et al., 2010
Probability of MT rescue at the KT	P_{res}	0.6	Fig. 4 B in Tanaka et al., 2007

This table shows the values of parameters used in the Spatiotemporal simulation of KT–MT interaction section of Materials and methods. In the Source of the value column, we mention relevant figures in the cited references where we can.

Table S2 is a separate Excel file showing the genotypes of yeast strains used in this study.

References

Clarke, P.R., and C. Zhang. 2008. Spatial and temporal coordination of mitosis by Ran GTPase. *Nat. Rev. Mol. Cell Biol.* 9:464–477. http://dx.doi.org/10.1038/nrm2410

Gandhi, S.R., M. Gierliński, A. Mino, K. Tanaka, E. Kitamura, L. Clayton, and T.U. Tanaka. 2011. Kinetochore-dependent microtubule rescue ensures their efficient and sustained interactions in early mitosis. *Dev. Cell.* 21:920–933. http://dx.doi.org/10.1016/j.devcel.2011.09.006

Haruki, H., J. Nishikawa, and U.K. Laemmli. 2008. The anchor-away technique: rapid, conditional establishment of yeast mutant phenotypes. *Mol. Cell.* 31:925–932. http://dx.doi.org/10.1016/j.molcel.2008.07.020

Kalinina, I., A. Nandi, P. Delivani, M.R. Chacón, A.H. Klemm, D. Ramunno-Johnson, A. Krull, B. Lindner, N. Pavin, and I.M. Tolić-Nørrelykke. 2013. Pivoting of microtubules around the spindle pole accelerates kinetochore capture. *Nat. Cell Biol.* 15:82–87. http://dx.doi.org/10.1038/ncb2640

Kitamura, E., K. Tanaka, Y. Kitamura, and T.U. Tanaka. 2007. Kinetochore microtubule interaction during S phase in *Saccharomyces cerevisiae*. *Genes Dev.* 21:3319–3330. http://dx.doi.org/10.1101/gad.449407

Kitamura, E., K. Tanaka, S. Komoto, Y. Kitamura, C. Antony, and T.U. Tanaka. 2010. Kinetochores generate microtubules with distal plus ends: their roles and limited lifetime in mitosis. *Dev. Cell.* 18:248–259. http://dx.doi.org/10.1016/j.devcel.2009.12.018

Natsume, T., C.A. Müller, Y. Katou, R. Retkute, M. Gierliński, H. Araki, J.J. Blow, K. Shirahige, C.A. Nieduszynski, and T.U. Tanaka. 2013. Kinetochores coordinate pericentromeric cohesion and early DNA replication by Cdc7-Dbf4 kinase recruitment. *Mol. Cell.* 50:661–674. http://dx.doi.org/10.1016/j.molcel.2013.05.011

Nishimura, K., T. Fukagawa, H. Takisawa, T. Kakimoto, and M. Kanemaki. 2009. An auxin-based degron system for the rapid depletion of proteins in nonplant cells. *Nat. Methods.* 6:917–922. http://dx.doi.org/10.1038/nmeth.1401

Tanaka, K., N. Mukae, H. Dewar, M. van Breugel, E.K. James, A.R. Prescott, C. Antony, and T.U. Tanaka. 2005. Molecular mechanisms of kinetochore capture by spindle microtubules. *Nature.* 434:987–994. http://dx.doi.org/10.1038/nature03483

Tanaka, K., E. Kitamura, Y. Kitamura, and T.U. Tanaka. 2007. Molecular mechanisms of microtubule-dependent kinetochore transport toward spindle poles. *J. Cell Biol.* 178:269–281. http://dx.doi.org/10.1083/jcb.200702141
